# Bootstrap approach to validate the performance of models for predicting mortality risk temperature in Portuguese Metropolitan Areas

**DOI:** 10.1186/s12940-019-0462-x

**Published:** 2019-03-29

**Authors:** Mónica Rodrigues, Paula Santana, Alfredo Rocha

**Affiliations:** 1 Centre of Studies on Geography and Spatial Planning, Department of Geography and Tourism, University of Coimbra, Coimbra, Portugal; 2 Centre for Environmental and Marine Studies, Department of Physics, University of Aveiro, Aveiro, Portugal

**Keywords:** Diseases of the circulatory system, Extreme temperatures, Distributed lag non-linear model (DLNM), Bootstrap approach, Model validation, Portugal

## Abstract

**Background:**

There has been increasing interest in assessing the impacts of extreme temperatures on mortality due to diseases of the circulatory system. This is further relevant for future climate scenarios where marked changes in climate are expected. This paper presents a solid method do identify the relationship between extreme temperatures and mortality risk by using as predictors simulated temperature data for cold and hot conditions in two urban areas in Portugal.

**Methods:**

Based on the mortality and meteorological data from Porto Metropolitan Area (PMA) and Lisbon Metropolitan Area (LMA), a distributed lag nonlinear model (DLNM) was implemented to estimate the temperature effects on mortality due to diseases of the circulatory system. The performance of the models was validated via bootstrapping approaching by creating resamples with replacement from the validating data. Bootstrapping was also used to identify the best candidate model and to evaluate the sensitivity of the spline functions to the exposure-lag-response relationship.

**Results:**

It is found that the model is able to reproduce the temperature-related mortality risk for two metropolitan areas. Temperature previously simulated by climate models is useful and even better than observed temperature. Although, the biases in predictions in both metropolitan areas are low, mortality risk predictions in PMA are more accurate than in LMA. Using parametric bootstrapping, we found that the overall cumulative association estimated under different bi-dimensional exposure-lag-response relationship are relatively stable, especially for the model selected by Quasi-Akaike Information Criteria (QAIC). Exposure to summer temperature conditions is best related to mortality risk. The association between winter temperature and mortality risk is somewhat less strong.

**Conclusions:**

The use of QAIC to choose from several candidate models provides valid predictions and reduced the uncertainty in the estimated relative risk for circulatory disease mortality. Our findings can be applied to better understand the characteristics and facilitate the prevention of circulatory disease mortality in Porto and Lisbon Metropolitan Areas, namely if we consider the actual context of climate change.

**Electronic supplementary material:**

The online version of this article (10.1186/s12940-019-0462-x) contains supplementary material, which is available to authorized users.

## Background

There is an extensive literature describing temperature-mortality associations and the extreme temperatures are one of leading causes of death in many countries [1–6]. Portugal has been identified as one of the countries in Europe with excess mortality during the winter [7, 8]. The Eurowinter Group [7] evaluated the impact of air temperature on mortality across some European countries and found smaller increases in cold mortality in northern European countries than in Greece (southern European countries). During the summer months, mortality is lower; however, sharp mortality peaks have been registered in years with heatwaves. This fact is reported by several studies, thus proving the association between extreme temperatures and cardiovascular and respiratory mortality [9, 10].

In Portugal, developing and publishing Cold Weather [11, 12] and Heatwave Plans [13], as formulated by the national government, have an important role in devising preventive measures so as to face health risks associated with extreme weather [14]. Nevertheless, in case of frequent extreme temperatures that may occur in the short- and long-term, this system does not suffice on its own. In extreme weather events, the event itself must be predicted, as well as its possible impacts on population health. Many climate models do not simulate urban climates, meaning that future heat-related mortality within cities is likely to be underestimated [14]. A study [15] reported that, during a severe heat wave in the UK, future mortality, estimated by adding temperature changes from a regional climate model to the modelled present-day temperatures, was notably higher when urban temperatures were used, in comparison with rural values.

Including projections from Global Climate Models (GCM) and Regional Climate Models (RCM) in national alert systems would be not only an asset in terms of short- and long-term prediction of these extreme events, but also a valuable tool for mitigating the effects that climate change has had on the regions under study, as well as on Portugal as a whole. In both GCM and RCM climate projections, a historical period is defined [16, 17]. Studies suggest [17, 18] that correction factors are derived by comparing the RCM output with observed weather variables in the control period and then applied to RCM output for future climate. The methodology validation based on a bootstrapping approach is found in different studies. A study developed for the Northern Eurasian Earth Science Partnership Initiative uses a bootstrapping approach that samples the full historical record to test the fidelity of the downscaling [18]. This method was used to evaluate several characteristics of the temperature–mortality relationships. For example, to quantify uncertainty, an approximate bootstrap method was proposed to derive the empirical distribution of the minimum mortality temperature for 135 cities in the USA [19]. The same study suggested that the proposed method performs better even with a minimal level of prior knowledge, reducing the Bias and RMSE in point estimation and achieving near 95% coverage while shortening the length in interval estimation. Another study [20], developed for the 52 provincial capital cities in Spain, proposed an approximate parametric bootstrap estimator of confidence interval from a temperature–mortality shape, estimated by splines, and showed that uncertainty can be small or large, depending on the estimated association pattern that varies among cities.

The aim of this study is to develop metropolitan-specific distributed-lag models and a method of validation using climate simulations of daily temperatures performed with the regional Weather and Research Forecast (WRF) model forced with the global climate model MPI-ESM-LR. This study uses simulated temperature data and serves as a reference for subsequent studies to be performed with the same climate model to study the impact of future climate change scenarios on mortality risk in the region.

We consider for the study, metropolitan areas of greatest importance in Portugal namely, Lisboa and Porto. The selection was based on the fact that these two metropolitan areas gather the population, the principal service activities and many of them are ill-equipped for climate change adaptation when focusing health population.

## Methods

The geographical areas under study comprises the Portuguese Metropolitan Areas (PMA, Porto Metropolitan Area; and LMA, Lisbon Metropolitan Area) for which the daily mortality and meteorological series were collected for the period 1986–2005.

### Data sources

#### Mortality data

The daily deaths counts were provided by the Statistics Portugal. Mortality data were classified into the following categories using the International Statistical Classification of Diseases: Diseases of the Circulatory System (ICD – 9: 390-459; ICD-10: I00-I99).

#### Meteorological data

Observed daily-average, maximum and minimum temperatures data were obtained from the NOAA’s National Climatic Data Center (NCDC), for meteorological stations in each metropolitan area: 85790 Gago Coutinho (LMA) and 085450 Pedras Rubras (PMA). Temperature measurements in Kelvin were converted to degrees Celsius. The selection of meteorological stations was based in their high quality data and their climatic representativeness location.

We obtained daily temperatures (daily-average, maximum and minimum) for each metropolitan area from simulations previously performed with the WRF model v3.5 [21] for the time period 1986–2005. The simulated temperature data was obtained for model grid-point nearest to both urban areas. This model was used to dynamically downscale climate data from the Max Planck Institute for Meteorology Earth System Model (MPI-ESM-LR) to a high-resolution (9-km) climatic grid. The global climate model participated in the IPCC 5th Assessment Report [22]. The detailed explanation of these simulations and their validation for the Iberian Peninsula are presented in [23]. Various studies have used these simulations for other purposes [24–27].

Simulated climate may have systematic errors when compared to observations. With the purpose to minimize the differences between simulated and observational data, numerous studies [28–32] have applied bias correction to the simulations.

In this study, we used a method developed by [33], which enables the minimization of the systematic errors verified in the daily maximum and minimum temperature simulations, through a quantile-quantile calibration. The advantage of this approach is the correction of the complete distribution including the tails, which, in this case, comprises the correction of extreme temperatures. Bias correction of temperature for the 1986–2005 period was performed by projecting the distribution of the observed temperature onto the simulated temperature for the period [33].

### Statistical analysis

#### Modeling the temperature-mortality association

We fitted a Metropolitan Area-specific time-series quasi-Poisson regression models adjusting for season, long-term trend (Year), days of the week (DOW), holiday (HOY), population (POP) and meteorological variables. In this situation, the outcome, *Y*_*t*_ at a given time *t* may be explained in terms of past exposures *x*_*t −* ℓ_, with ℓ as the lag. The general model definition is

1 $$ g\left({\mu}_t\right)=\propto +{\sum}_{j=1}^J{s}_j\left({x}_{ij}{\beta}_j\right)+{\sum}_{k=1}^K{\gamma}_k{u}_{tk} $$

where *μ*_*t*_ ≡ *E*(*Y*_*t*_), and *Y*_*t*_ daily deaths by CS with t = 1,…,n, assumed arise from an over-dispersed Poisson distribution. The function *s*_*j*_ specify the relationship between variables *x*_*j*_ and the nonlinear exposure-response curve, defined by the parameter vectors *β*_*j*_. That is a non-linear and delayed effects of a predictor will be modeled through the functions *s*_*j*_ which define the relationship along the two dimensions of predictor and lags. The variables *u*_*k*_ include other predictors with linear effects specified by the related coefficients *γ*_*k*_. To eliminate the potential confounding effects, we included the following covariates in our time-series regression models.

The Metropolitan Area-specific model is given as:

2 $$ \log \left[E\left({Y}_{it}\right)\right]=\alpha +{\delta}_1{DOW}_t+{\delta}_2{HOY}_t+{\delta}_3{Pop}_t+ ns\left({Date}_t\right)+{cb}_{temp}\left({temp}_t,\dots, {temp}_{t-30}\right) $$

where *E*(*Y*_*it*_) denotes the daily number of deaths on day *t*; *t* refers to the day of the observation; *α* is the intercept; ns() denotes the cubic smoothing spline; DATE_t_ is the day of calendar time on day *t*, with 7–9 degrees of freedom per year [1]; DOW_t_ is the day of the week on day *t*, HOY_t_ is holiday or not on day *t,* POP_t_ is the population [11].

The cb (Temperature, lag) is a *cross-basis* matrix obtained by applying to temperature lag-response exposure; here lag refers to the maximum 30-lag days. That is, we modeled the non-linear and delayed exposure-lag-response relationship between temperature and mortality with a distributed lag non-linear model applying a bi-dimensional cross-basis spline function describing the dependency along the temperature range and its 30 days of lag dimension [34, 35].

Briefly, the *cross-basis* function *s*(*x*, *t*) is defined by:

3 $$ \kern8.25em s\left(x,t\right)\approx \sum \limits_{l={l}_0}^Lf.\omega \left({x}_{t-l},l\right) $$

with bi-dimensional function, *f*. *ω*(*x*_*t* − *l*_, *l*) composed of two marginal functions *f*(*x*) and *ω*(*l*) representing the smooth function for exposure-response and lag-response function, respectively.

Therefore, for a given time *t*, the *cross-basis* parameterization can be re-expressed as:

4 $$ {w}_{x,t}^T\eta =\sum \limits_{l={l}_0}^L{\omega}_{temp_{t-l},l} $$

This *overall cumulative* association is composed of the sum of contributions *β*_*x*, *l*_ from exposures *temp*_*t* − *lo*_, … , *temp*_*t* − *L*_ experienced within the 30-lag period.

#### Prediction for cumulative exposure-response association

For each Metropolitan Area, predictions for overall cumulative exposure-response risk associations were derived from the parameter estimates from the training data for varying temperature values. The analysis was carried out for both observed and predicted data as follows:

1. The observed data for the period of 1986–2000 was used as training data and validated using the observed test data for the period 2001–2005.
2. The simulated data for the period of 1986–2000 was used as training data and validated using the simulated test data for period 2001–2005.

Each model was built considering the minimum and mean temperatures for winter (December, January, February and March), while maximum and mean temperatures were used for summer (June, July, August and September). The predictions for the cumulative exposure–lag–response association were derived from the parameter estimates from the DLNM regression models of Eq. 2 for specific covariates values, eg. varying temperature values, lags and other predictors.

**Fig. 1:**
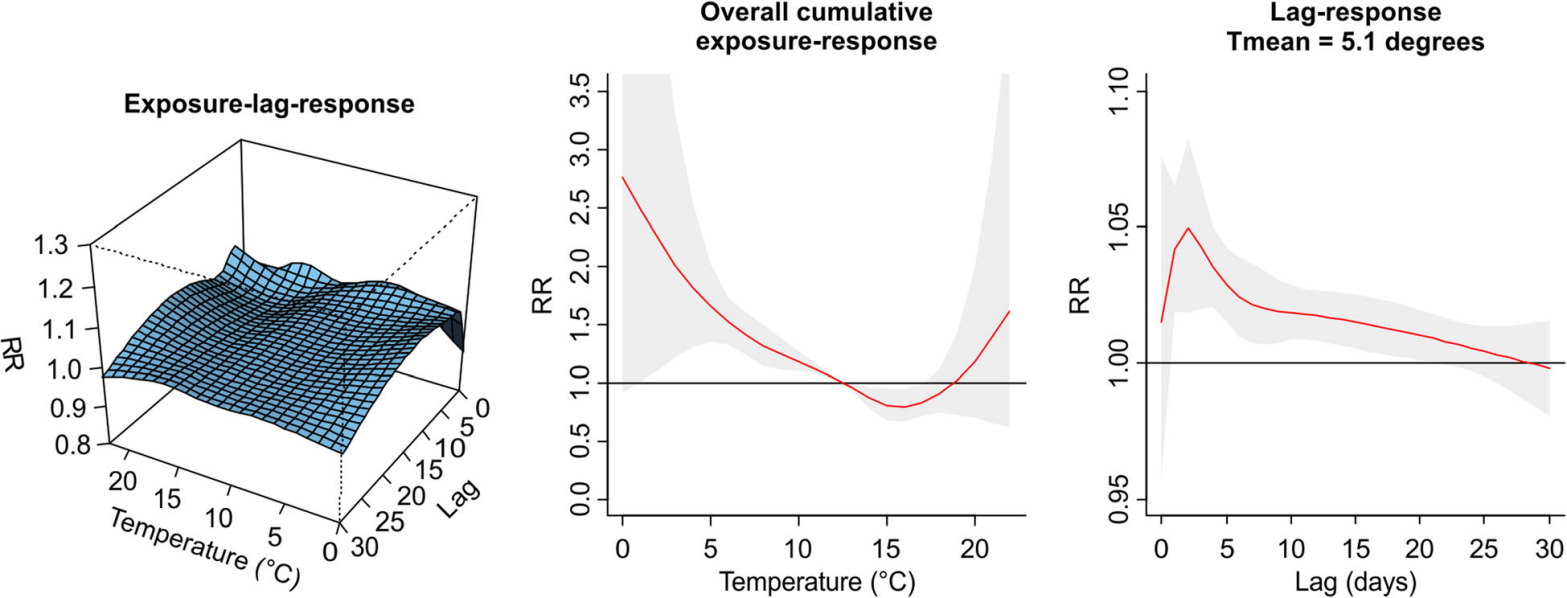
Exposure -lag-response, overall cumulative exposure - response, and lag-response for observed data in WINTER for Porto Metropolitan Area (PMA), during 1986–2000, using mean temperature (Tmean)

**Fig. 2:**
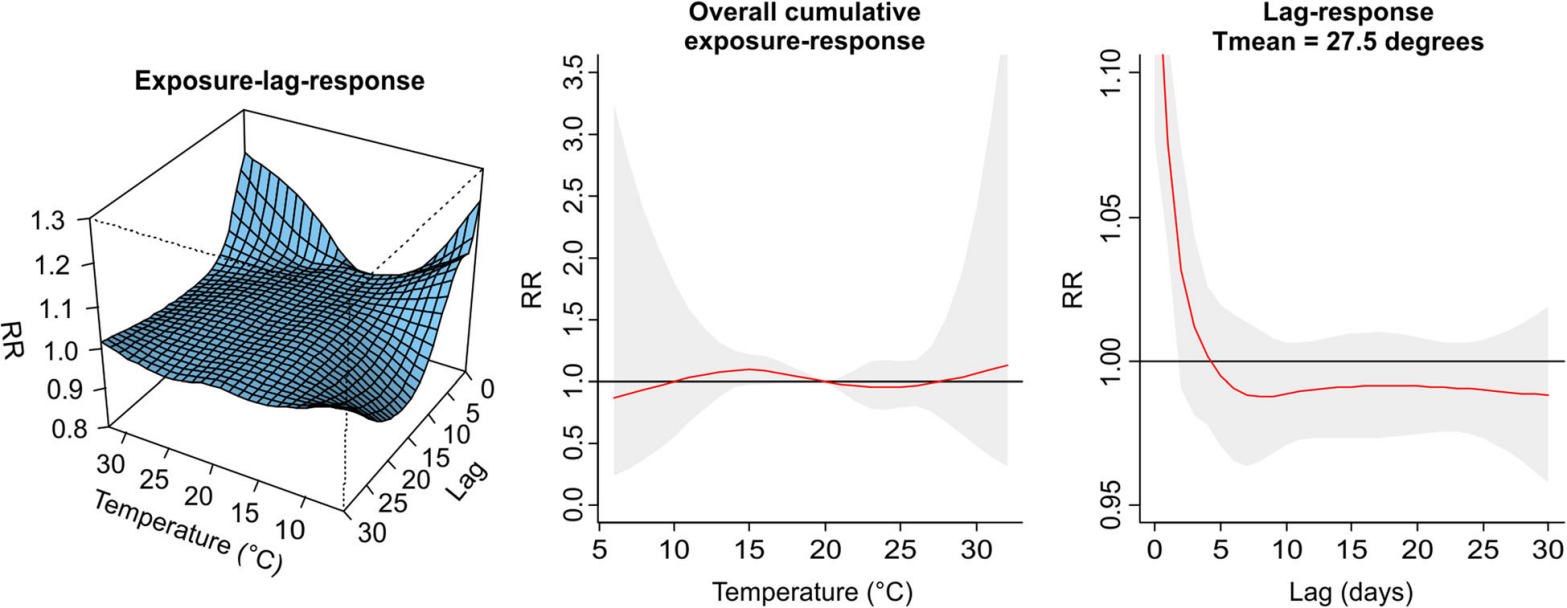
Exposure-lag-response, overall cumulative exposure - response, and lag-response for observed data in SUMMER for Porto Metropolitan Area (PMA), during 1986–2000, using mean temperature (Tmean)

**Fig. 3:**
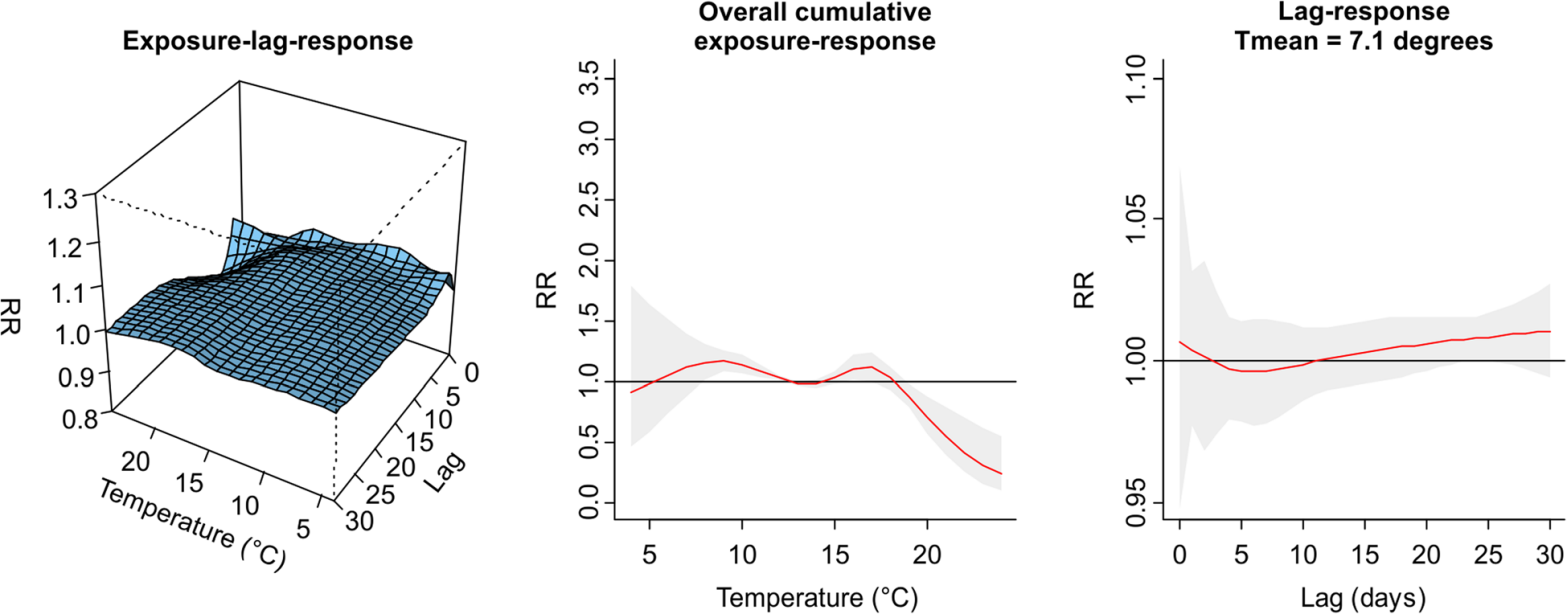
Exposure -lag-response, overall cumulative exposure - response, and lag-response for observed data in WINTER for Lisbon Metropolitan Area (LMA), during 1986–2000, using mean temperature (Tmean)

**Fig. 4:**
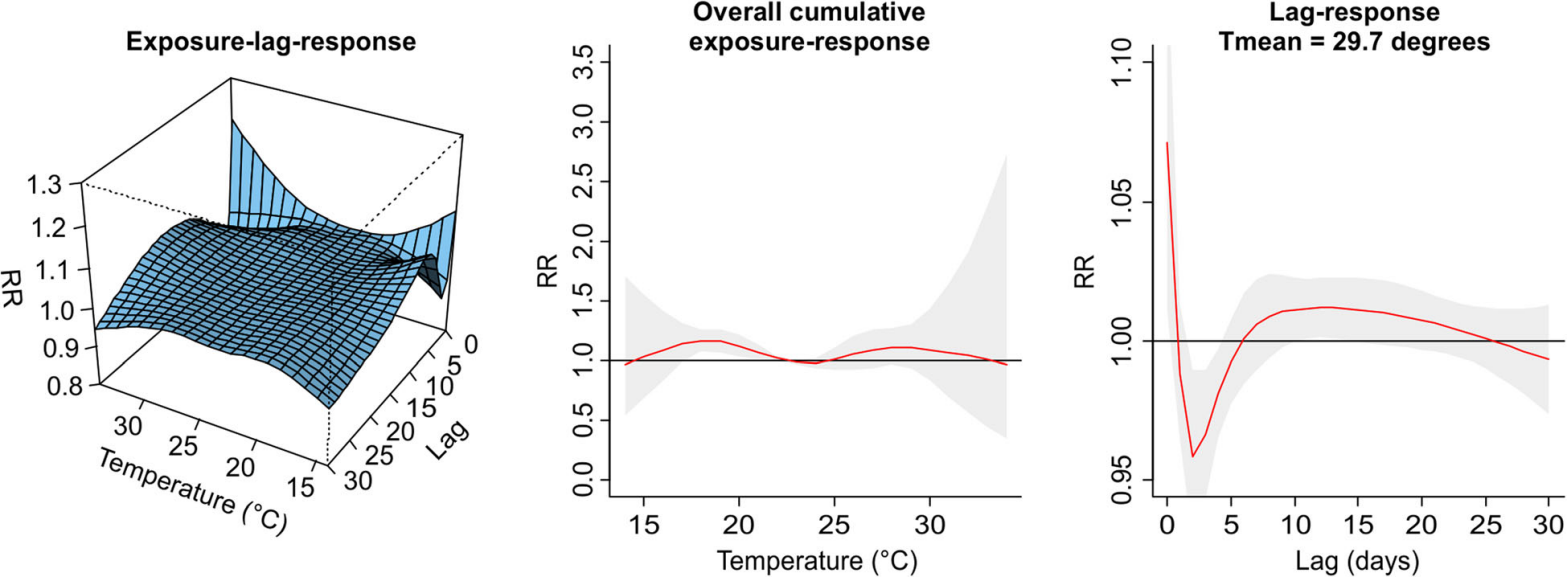
Exposure -lag-response, overall cumulative exposure - response, and lag-response for observed data in SUMMER for Lisbon Metropolitan Area (LMA), during 1986–2000, using mean temperature (Tmean)

**Fig. 5:**
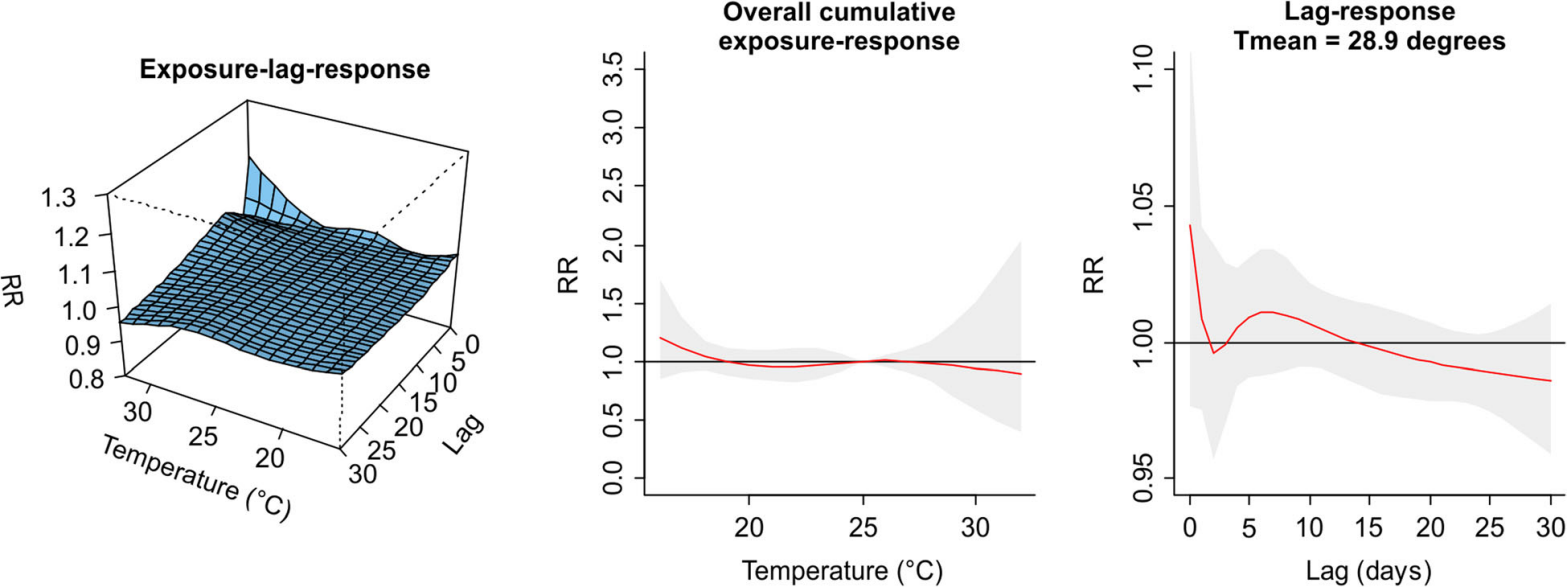
Exposure -lag-response, overall cumulative exposure - response, and lag-response for simulated data in SUMMER for Lisbon Metropolitan Area (LMA), during 1986–2000, using mean temperature (Tmean)

**Fig. 6:**
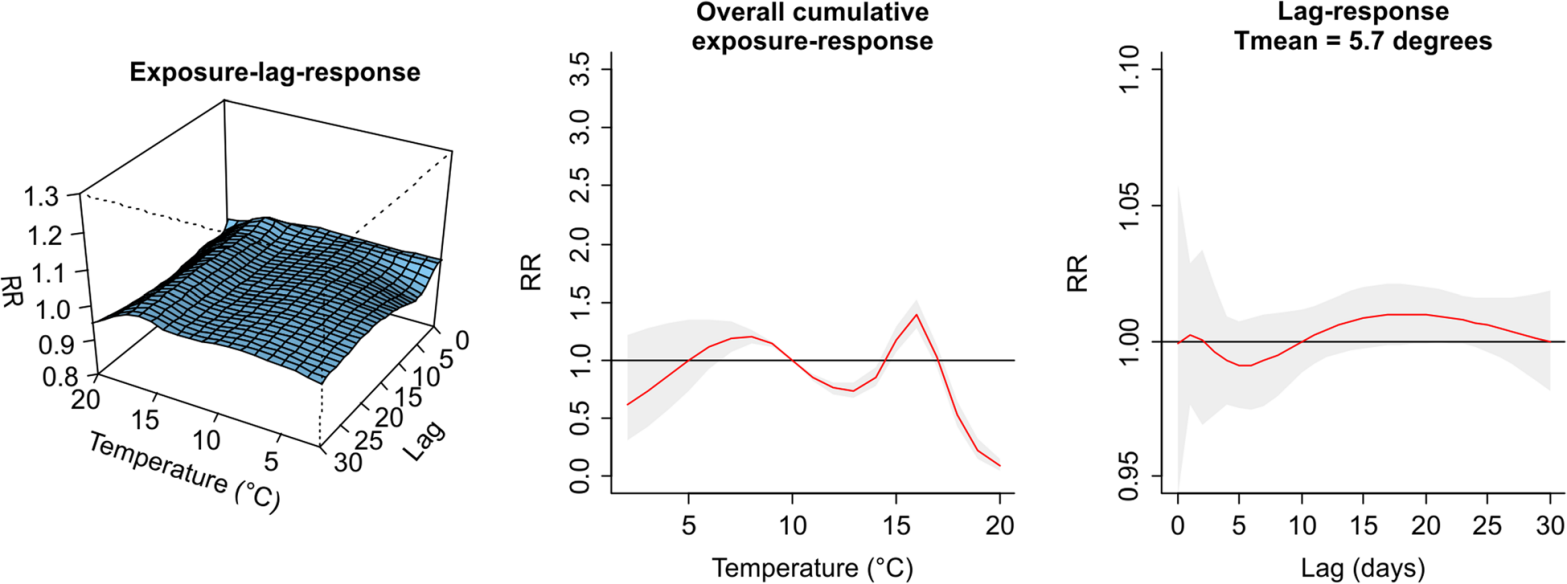
Exposure -lag-response, overall cumulative exposure - response, and lag-response for simulated data in WINTER for Lisbon Metropolitan Area (LMA), during 1986–2000 using mean temperature (Tmean)

**Fig. 7:**
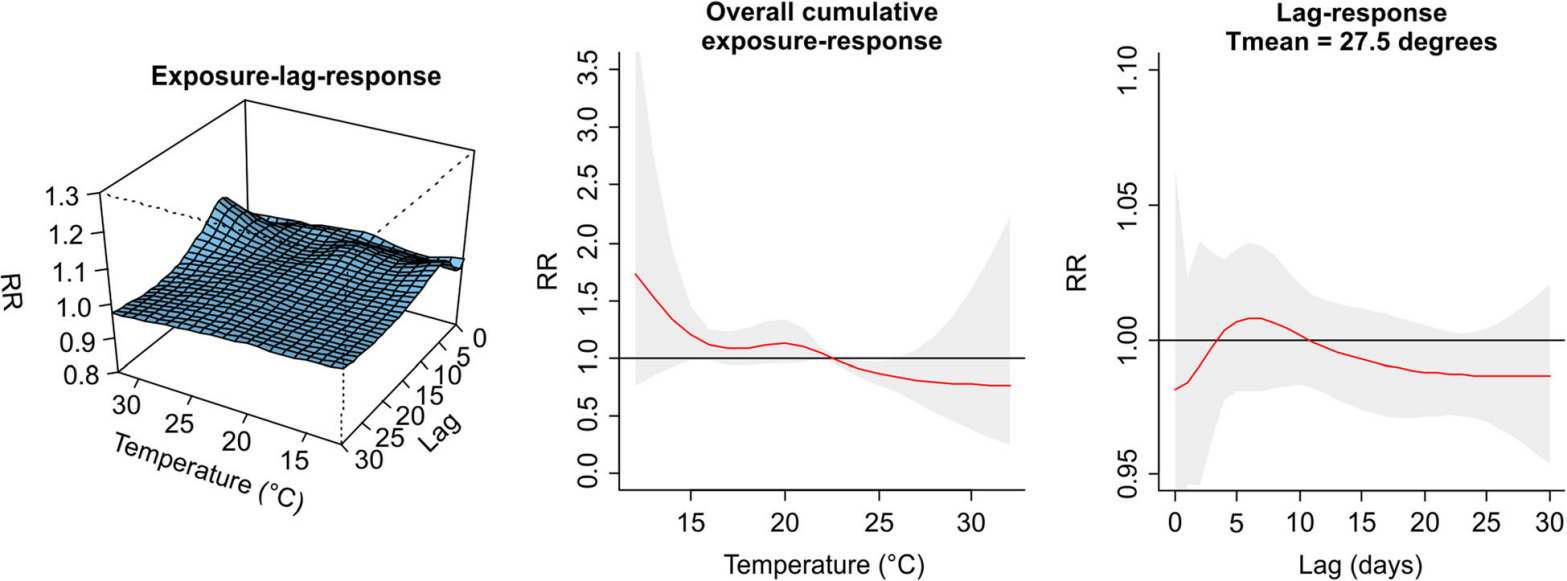
Exposure -lag-response, overall cumulative exposure - response, and lag-response for simulated data in SUMMER for Porto Metropolitan Area (PMA), during 1986–2000 using mean temperature (Tmean)

**Fig. 8:**
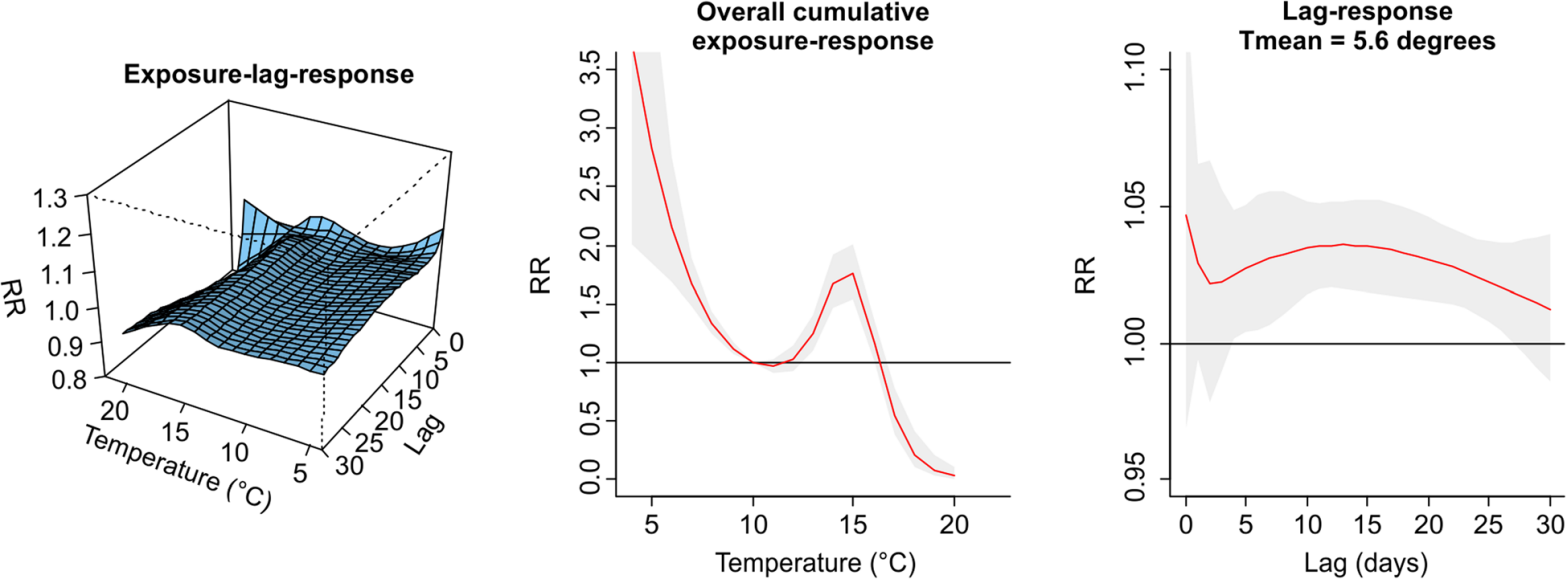
Exposure -lag-response, overall cumulative exposure - response, and lag-response for simulated data in WINTER for Porto Metropolitan Area (PMA), during 1986–2000 using mean temperature (Tmean)

The values plotted in Figs. 1, 2, 3, 4, 5, 6, 7 and 8 are derived based on different settings of the regression variables. For example, in a panel of the figures we keep the temperature values at 1st percentile for temperatures in the winter and 99th percentile for the summer data to estimate the relative risk. The specific values are presented in Tables 2 & 3. For the 3D prediction for cumulative exposure-response association in left panel of Figs. 1, 2, 3, 4, 5, 6, 7 and 8, various temperature values were combined with several lag-days. The values were derived from a combination of multi-variables, lag-days as well as various temperature.

### Sensitivity analysis for model parameters

We carried out a sensitivity analysis to investigate our model choices. The model selection for the number of knots and position, number of dfs and smooth function for exposure-response and lag-response function are based on modified Akaike information criteria for models with overdispersed data Quasi-AIC [35]. See the appendix Additional file 1: Table S1 for the different formulation of the 48 candidate models.

### Model validation and evaluation of performance

Firstly, we validate the estimates of the risk contributions corresponding to a specific temperature exposure intensity at different lag, ${\beta}_{temp_{t-l},l}$ without resampling. We computed different estimated exposure–lag–response risk association ${\beta}_{temp_{t-l},l}$ at the varying exposure and lags by DLNM model (eq. 2) with a cross-basis defined in (eq. 3).

We derive root mean square error (RMSE) and mean absolute deviation (MAD) as follow:

5 $$ \mathrm{RMSE}=\frac{\ \sqrt{\frac{1}{n}\sum {\left({\widehat{\beta}}_i-{\beta}_i\right)}^2}}{\beta_i/n}\ \mathrm{and}\ \mathrm{MADs}=\frac{\left|\raisebox{1ex}{$\sum \left({\widehat{\beta}}_i-{\beta}_i\right)$}\!\left/ \!\raisebox{-1ex}{$n$}\right.\right|}{\beta_i/n} $$

where ${\widehat{\beta}}_i$ is estimated from the training data (1986–2000) and *β*_*i*_ is estimated from the test data (2001–2005).

Secondly, the performance of the models was validated via bootstrapping approaching by creating resamples with replacement from the validating data (2001–2005), of the same size, then use the model to predict the values of the of original data. Finally, the root mean squared error between the predicted and observed data was computed. We compute the predicted number of deaths, then repeat this step 50 times for resampled data sets. That is, for each data set (*i = 1,…, 50*), the prediction of deaths for the exposure–lag–response association is estimated by DLNM model (eq. 2) with a cross-basis defined in (eq. 3).

The error of the predicted the number of deaths from the random sample relative to the actual value is estimated by root mean squared error:

6 $$ \mathrm{RMSE}=\sqrt{\frac{1}{n}\sum {\left({Y}_{obs,i}-{Y}_{pred,i}\right)}^2} $$

where the observed deaths in simulation “i” and predicted deaths in simulation “i” are designated by *Y*_*obs*, *i*_ and *Y*_*pred*_, *i*, respectively.

The average error is estimated from the differences between the RMSE for the simulated data and that of the original data.

Lastly, adopting the strategy of Gasparrini et al. [35], we carried out a simulation study to assess the performance of DLNM to investigating the variation in the overall cumulative effect, *β*_*i*_ due to the specification of cross-basis spline function for exposure-lag-response specification and degrees of freedom for day of calendar to capture trend and seasonality in the data. The bootstrap samples were developed as follows:

1. Generate m = 500 bootstrap samples of size n_s_ subjects (n_s_ = 2000) each.
2. For each simulated sample, the overall cumulative exposure-lag-response association is estimated by DLNM (2) under each of scenarios:
  Define the scenarios for cross-basis spline functions *f*(*x*) and *ω*(*l*). The exposure-response function, *f*(*x*) was specified as a simple linear term or quadratic B-splines with 0, 1 or 2 knots placed at equal distance in the dimension of the predictor while the lag-response function *ω*(*l*) was specified as a simple constant term with 1 df or quadratic B-splines with intercept and 0, 1 or 2 knots equally placed.
  The dfs for spline for “Date” to account for trends and seasonality were set at 7–9.

In total, s = 48 models were evaluated with total df ranges 1 to 20 (Additional file 1: Table S1).

We estimate the bootstrap percent bias, the coverage probability and relative root mean square error (RMSE) of the overall cumulative effect, *β*. These indices are given by the equations below:

7 $$ \mathrm{Bias}=\frac{\left|\raisebox{1ex}{$\sum \left({\widehat{\beta}}_{c,i}-{\beta}_{c,i}\right)$}\!\left/ \!\raisebox{-1ex}{$m$}\right.\right|}{\sum {\beta}_{c,i}/m}\times 100 $$

8 $$ \mathrm{Coverage}\ \mathrm{probability}=\sum I\left(|{\widehat{\beta}}_{c,i}-{\beta}_{c,i}|\Big)\le 1.96\bullet \sqrt{V\left({\widehat{\beta}}_{c,i}\right)}\right)/m $$

9 $$ \mathrm{RMSE}=\frac{\ \sqrt{\frac{1}{m}\sum {\left({\widehat{\beta}}_{c,i}-{\beta}_{c,i}\right)}^2}}{\sum {\beta}_{c,i}/m}\times 100 $$

Where I is an indicator function, ${\widehat{\beta}}_{c,i}$ is the estimated overall cumulative effect from the best fitting model selected by QAIC and ${\widehat{\beta}}_{c,i}$ is the summary effect at each *i*^*th*^ iteration.

All the data analyses were conducted in R software (version 3.4.0, R Project for statistical Computing, http://www.r-project.org), using the package “dlnm” [36].

## Results

### Data description

This study is based on observed/simulated number of deaths and exposure to temperature in PMA (Porto Metropolitan Area) and LMA (Lisbon Metropolitan Area), from 1986 to 2005. Summary statistics of mortality are shown in Table 1. The overall average number of deaths were 14.2 and 29.1 for PMA and LMA, respectively from 1986 to 2000. The average number of deaths for PMA and LMA in winter of the same period were 17.4 and 36.0 respectively.

**Table 1:** Region-specific summary deaths statistics, for Summer and Winter months during 1986–2005, for Porto Metropolitan Area (PMA) and Lisbon Metropolitan Area (LMA), Portugal

Metropolitan Area/Study Period	Summer Mean (SD)	Winter Mean (SD)	Overall Mean (SD)
PMA 1986–2000	11.9 (3.6)	17.4 (5.1)	14.2 (4.9)
LMA 1986–2000	24.1 (6.0)	36.0 (8.2)	29.1 (8.4)
PMA 2001–2005	14.3 (11.4)	9.2 (6.4)	11.7 (9.8)
LMA 2001–2005	23.3 (6.2)	34.8 (8.2)	27.8 (8.4)

Table 2 presents some descriptive statistics for observed temperature. For winters of 1986–2000 and 2001–2005 in LMA, the average and 1st percentile of daily mean and minimum temperatures are shown. The average daily mean temperatures in LMA were 12.8 °C and 11.3 °C in 1986–2000 and 2001–2005, respectively compared to 11.2 °C and 11.3 °C in the same period in PMA. Similarly, the average daily minimum temperature and 1st percentile daily minimum temperature in LMA 1986–2000 were 9.5 °C and 3.9 °C, respectively and 9.1 °C and 2.1 °C, respectively in 2001–2005. In the PMA the average daily minimum temperature was 7.2 °C and 7.2 °C in 1986–2000 and 2001–2005, respectively. Similar patterns can be observed in the summer period for both Metropolitan Areas in 1986–2000 and 2001–2005.

**Table 2:** Descriptive statistics for observed temperature values in Porto Metropolitan Areas (PMA) and Lisbon Metropolitan Areas (LMA), used in the models for the period 1986–2000 and validating period 2001–2005 together with the estimated relative risks (RRs) values at averages, 1st and 99th percentiles temperature values

WINTER								
Metropolitan Area/Study Period	Ave. Tmean	RR (95% CI)	1st Tmean	RR (95% CI)	Ave. Tmin	RR (95% CI)	1st Tmin	RR (95% CI)
PMA 1986–2000	11.2	1.10 (1.06–1.13)	5.1	1.63 (1.35–1.97)	7.2	0.81 (0.75–0.86)	− 0.3	1.34 (1.07–1.67)
LMA 1986–2000	12.8	0.99 (0.97–0.99)	7.1	1.11 (0.89–1.38)	9.5	1.02 (1.01–1.02)	3.9	1.75 (1.54–1.99)
PMA 2001–2005	11.3	1.09 (1.05–1.13)	5.4	1.98 (1.19–3.29)	7.2	1.02 (1.01–1.03)	− 0.3	1.52 (0.98–2.38)
LMA 2001–2005	11.3	0.93 (0.89–0.96)	4.6	0.95 (0.66–1.36)	9.1	1.03 (1.00–1.05)	2.1	3.03 (2.16–4.25)
SUMMER								
Metropolitan Area/Study Period	Ave. Tmean	RR (95% CI)	99th Tmean	RR (95% CI)	Ave. Tmax	RR (95% CI)	99th Tmax	RR (95% CI)
PMA 1986–2000	19.1	1.02 (0.99–1.05)	27.5	1.00 (0.72–1.39)	23.5	1.03 (0.89–1.18)	35.0	0.91 (0.58–1.42)
LMA 1986–2000	22.2	1.01 (1.00–1.02)	29.7	1.10 (0.87–1.38)	22.6	0.89 (0.79–1.01)	33.1	1.03 (0.79–1.33)
PMA 2001–2005	19.4	1.07 (0.91–1.27)	28.7	1.09 (0.51–2.34)	23.7	1.02 (0.89–1.17)	35.5	0.91 (0.56–1.44)
LMA 2001–2005	19.5	1.12 (1.03–1.22)	27.9	1.10 (0.82–1.46)	27.7	0.97 (0.92–1.02)	38.4	1.01 (0.69–1.45)

### Model selection

We present the assessment of model parameter choices in Additional file 1: Table S1 for 48 candidate models. The best fitted model for temperature-mortality association according to QAIC is Model 11. This model described the mean daily temperature delayed effects (lag 0–30) by quadratic B-spline function for both exposure-response and lag-response with a total df of 12 and knot placed at 13.5 for temperature and 15 for lags. The model also include the term “Date” described by a natural cubic spline function to control for long term temporal trend and seasonality with 8 dfs generated per year of study.

### Temperature-mortality association

Results for DLNM (Model 11), assuming a nonlinear (quadratic B-spline) temperature exposure–death relationship are presented in Table 2. These results rely on a bidimensional representation of the exposure–lag–response association as depicted in Figs. 1, 2, 3, 4, 5, 6, 7 and 8 over the range of the exposure and the lag days, show the predicted relative risk (RR) surfaces from the model in eq. 1, for various temperature ranges and 0–30 lags.

For the winter season in PMA (1986–2000) and considering exposure to average daily observed temperature (Fig. 1, left), the exposure–lag–response show an initial increase in RR along lags, peaking at approximately 3 days after the exposure, and then decreasing and apparently disappearing after about 15 days, independent of the exposure levels. The right panel of Fig. 1 shows the associated risk composing the lag-response curve at 1st percentile of the daily mean temperature (5.1 °C). The estimated RR peaks at lag 3 and the risk disappears 6–10 days. This indicates that risk of deaths is highest after a 3 days (1–3 lag days) cumulative exposure to temperature at 5.1 °C. The relationship between temperature exposure (Tmean) and risk of deaths indicate a nonlinear temperature-mortality dependency (middle panel of Fig. 1). This part of the figure shows the predicted risk for different exposure intensities on the same day. The RR was highest at extreme cold temperature (1–5 °C) and decreases steadily. The estimated risk at an exposure of 5.1 °C (1st percentile Tmean) was RR of 1.63 (95% CI: 1.35–1.97) indicating a high risk of death at that temperature (Table 2).

The results for PMA in the summer period presented in Table 2 and displayed in Fig. 2 is completed different from what was observed in winter. The middle panel of Fig. 2 is almost flat indicating that the effect of daily mean temperatures in the summer were not significantly associated with risk of death.

Figures 3 and 4 presented the exposure-lag response association for LMA in winter and summer respectively. Figure 3 indicates that generally exposure to daily mean temperature has no significant effect on the risk of death at average temperature of 12.39 °C with lower RR of 0.99, (95% CI: 0.97–0.99) for the period 1986–2000. Similarly, in 2001–2000 there was a lower associated risk at daily mean temperature exposure of 11.39 °C with RR = 0.93 (95% CI: 0.89–0.96). There was an associated risk of 1.10 (95% CI: 1.00–1.02) in the summer period 1986–2000 and 1.12 (95% CI: 1.03–1.22) in the summer period 2001–2005 for average daily mean temperatures of 22.27 °C and 19.51 °C respectively. For this and other observed temperature values see Table 2 and Additional file 2: Figure S1, Additional file 3: Figure S2, Additional file 4: Figure S3, and Additional file 5: Figure S4.

Figures 5, 6, 7 and 8 present the exposure-lag-response for the simulated data for winter and summer during period 1986–2000 and 2001–2005. The descriptive summaries for temperature and the associated risk of deaths are displayed in Table 3. Summary statistics for temperatures in LMA are generally higher than PMA. Relative risks are higher and significant for 1st percentile minimum temperatures, for instance, in PMA the associated risk at a 1st percentile minimum temperature exposure of 0 °C for 1986–2000 was RR = 2.24, (95% CI: 1.69–2.97) while at − 2 .2 in 2001–2005 was RR = 4.71 (95% CI: 2.51–8.82).

**Table 3:** Descriptive statistics for simulated temperature values in Porto Metropolitan Area (PMA) and Lisbon Metropolitan Area (LMA), Portugal, used in the models for the period 1986–2000 and validating period 2001–2005 together with the estimated relative risks (RRs) values at averages, 1st and 99th percentiles temperature values

	WINTER							
Metropolitan Area/Study Period	Ave. Tmean	RR (95% CI)	1st Tmean	RR (95% CI)	Ave. Tmin	RR (95% CI)	1st Tmin	RR (95% CI)
PMA 1986–2000	11.3	0.97 (0.91–1.05)	5.6	2.39 (1.75–3.28)	7.7	1.00 (0.91–1.11)	0.0	2.24 (1.69–2.97)
LMA 1986–2000	12.4	0.74 (0.67–0.81)	5.7	1.09 (0.87–1.35)	9.8	1.00 (0.99–1.01)	3.4	1.55 (1.34–1.79)
PMA 2001–2005	10.4	0.99 (0.97–1.01)	4.6	1.97 (1.68–2.29)	6.7	0.97 (0.91–1.03)	−2.2	4.71 (2.51–8.82)
LMA 2001–2005	11.5	0.80 (0.77–0.84)	5.5	0.93 (0.67–1.28)	9.2	1.06 (1.03–1.09)	2.9	1.14 (0.77–1.69)
SUMMER								
Metropolitan Area/Study Period	Ave. Tmean	RR (95% CI)	99th Tmean	RR (95% CI)	Ave. Tmax	RR (95% CI)	99th Tmax	RR (95% CI)
PMA 1986–2000	19.2	1.12 (0.95–1.32)	27.5	0.81 (0.57–1.12)	23.7	1.11 (0.97–1.26)	34.8	0.82 (0.58–1.16)
LMA 1986–2000	21.6	0.96 (0.83–1.11)	28.9	0.97 (0.73–1.30)	23.9	0.98 (0.90–1.08)	35.3	1.01 (0.75–1.35)
PMA 2001–2005	19.8	1.13 (0.96–1.33)	28.8	0.79 (0.50–1.23)	24.7	1.10 (0.97–1.24)	36.5	0.83 (0.48–1.40)
LMA 2001–2005	22.2	0.97 (0.86–1.09)	30.4	0.99 (0.82–1.21)	24.85	1.02 (0.87–1.20)	38.4	1.05 (0.68–1.62)

Figure 5 presents the exposure-lag-response curve for summer in 1986–2000 and 2001–2005. The middle panel revealed a flat line implying no association between temperature and death in the summer while in Fig. 6 for winter period in Lisbon, the associated risk for 1st percentile average daily mean temperature of 9.2 °C with RR = 1.06 (95% CI: 1.03–1.09). See Table 3 and Additional file 6: Figure S5, Additional file 7: Figure S6, Additional file 8: Figure S7 and Additional file 9: Figure S8 for the relative risks associated with other temperatures in winter and summer for the period 1986–2000 and 2001–2005.

Overall, one can say that, from the analysis of Tables 2 and 3, the temperature and the associated RR are similar when using observed and simulated temperature. The errors presented in Table 4 confirms this statement with small MAD and RMSE in all cases with the exceptions for LMA using observed Tmean, simulated Tmean and Tmin. Higher RR are obtained for extreme low and high temperatures. We can, therefore, be confident in using simulated temperature to estimate RR for the present climate and for future climate scenarios.

**Table 4:** Summary of statistics for model prediction relative to original model for Porto Metropolitan Area (PMA) and Lisbon Metropolitan Area (LMA), Portugal. Values in bold represent lower errors using simulated rather than observed temperature

WINTER				
Model based on	Validated with PMA/LMA 2001–2005 Using Tmean		Validated with PMA/LMA 2001–2005 Using Tmin	
	MAD	RSME	MAD	RSME
PMA 1986–2000 (Observed)	0.1565	0.0521	0.1854	0.0407
LMA 1986–2000 (Observed)	0.4367	0.4916	0.1183	0.0579
PMA 1986–2000 (Simulated)	0.2205	0.0856	0.1095	0.0447
LMA 1986–2000 (Simulated)	0.3041	0.3296	0.3040	0.3296
SUMMER				
Model based on	Validated with PMA/LMA 2001–2005 Tmean		Validated with PMA/LMA 2001–2005 Using Tmax	
	MAD	RSME	MAD	RSME
PMA 1986–2000 (Observed)	0.1215	0.0299	0.0033	0.00002
LMA 1986–2000 (Observed)	0.0612	0.0068	0.0922	0.0299
PMA 1986–2000 (Simulated)	0.0079	0.0002	0.0068	0.0002
LMA 1986–2000 (Simulated)	0.0202	0.0010	0.0005	0.0208

### Variability in temperature-mortality association and prediction

We present here the results from the assessment of temperature-mortality associations and predictions (Additional file 10: Figure S9, Additional file 11: Figure S10, Additional file 12: Figure S11, Additional file 13: Figure S12, Additional file 14: Figure S13, Additional file 15: Figure S14, Additional file 16: Figure S15 and Additional file 17: Figure S16 and Tables 4 & 5).

**Table 5:** Synthetic indices of relative bias (mean) of exposure–lag–response associations, for Lisbon Metropolitan Area (LMA) and Porto Metropolitan Area (PMA). Results from 50 simulations

Season	Temperature (°C)	Bias	
		LMA	PMA
Winter	Tmean	0.0103 (0.0071)	−0.0033 (0.0196)
	Tmin	0.0167 (0.0067)	−0.0057 (0.0167)
Summer	Tmean	0.0052 (0.0116)	−0.0127 (0.0224)
	Tmax	0.0076 (0.0104)	−0.0064 (0.0235)

A panel of Additional file 10: Figure S9, Additional file 11: Figure S10, Additional file 12: Figure S11, Additional file 13: Figure S12, Additional file 14: Figure S13, Additional file 15: Figure S14, Additional file 16: Figure S15 and Additional file 17: Figure S16 show the pattern of the estimated associated risks for models fitted with the training data (1986–2000) validated with data sets from 2001 to 2005. The performance of the models was assessed via eq. 4 for the overall cumulative effect, and then visually assessed via of Additional file 10: Figure S9, Additional file 11: Figure S10, Additional file 12: Figure S11, Additional file 13: Figure S12, Additional file 14: Figure S13, Additional file 15: Figure S14, Additional file 16: Figure S15 and Additional file 17: Figure S16. The figures show the associated risks for the validated data resembles the associated risks from the training data (1986–2000). Visual assessment of the Additional file 10: Figure S9, Additional file 11: Figure S10, Additional file 12: Figure S11, Additional file 13: Figure S12, Additional file 14: Figure S13, Additional file 15: Figure S14, Additional file 16: Figure S15 and Additional file 17: Figure S16 show no distinctive differences between associated risks in 1986–2000 and 2001–2005 for the winter and summer periods. For example, the risk of death based on observed 1st percentile daily mean winter temperature of 5.1 °C in PMA (1986–2000, see Additional file 10: Figure S9) was RR = 1.63, (95%CI: 1.35–1.97) similar to RR = 1.98, (95%CI: 1.19–3.29) in 2001–2005 based on daily mean winter temperature of 5.4 °C. In the same vein, we observed similarity in associated risks of deaths between 99th percentile daily mean summer temperature of 27.5 °C in 1986–2000 (RR = 1.00, 95% CI: 0.72–1.39) and 99th percentile daily mean summer temperature of 28.7 °C (RR = 1.09, 95%: 0.82–1.46) in 2001–2005.

The visual assessments of the Additional file 10: Figure S9, Additional file 11: Figure S10, Additional file 12: Figure S11, Additional file 13: Figure S12, Additional file 14: Figure S13, Additional file 15: Figure S14, Additional file 16: Figure S15 and Additional file 17: Figure S16 confirm to the values of RMSE and MAD reported in Table 4 indicating a good prediction of the effect of temperature on deaths. As shown in these figures, the curves are very close to each other indicting the models provide a good prediction. In particular, the graphs for the summer periods are almost exactly overlaying each other. Also, Table 4 confirms results shown in Tables 2 and 3 that small MAD and RMSE are observed in all cases with the exceptions for LMA using observed Tmean and using simulated Tmean and Tmin. Higher RR are obtained for extreme low and high temperatures.

**Fig. 9:**
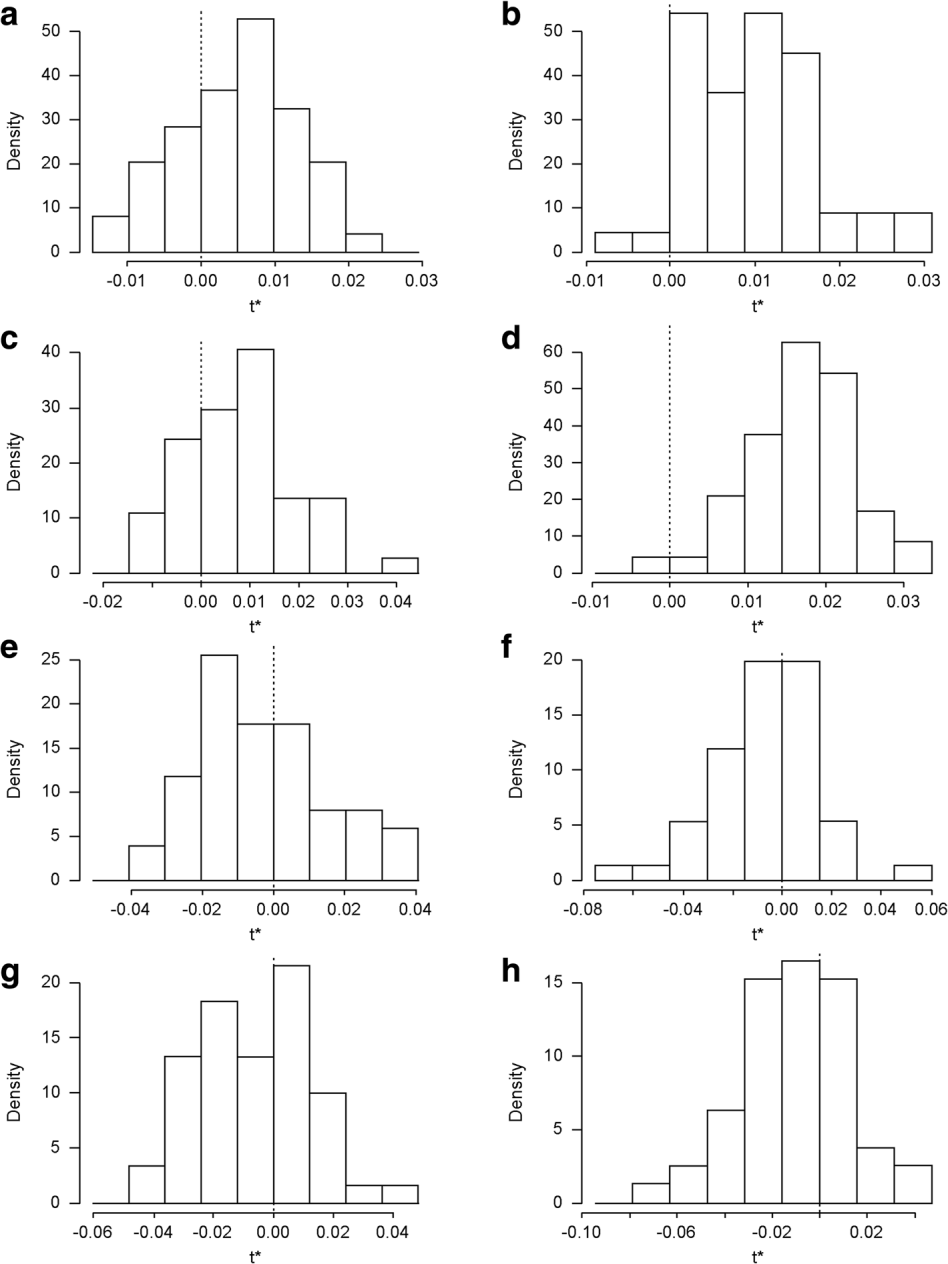
Histograms of model biases using data from 2001 to 2005. **a** Lisbon Metropolitan Area (LMA) Summer 2001–2005-Tmean (mean temperature); (**b**) LMA Winter 2001–2005-Tmean; (**c**) LMA Summer 2001–2005-Tmax (maximum temperature); (**d**) LMA Winter 2001–2005-Tmin (minimum temperature); (**e**) Porto Metropolitan Area (PMA) winter 2001–2005-Tmean; (**f**) PMA Summer 2001–2005-Tmean; (**g**) PMA winter 2001–2005-Tmin; (**h**) PMA Summer 2001–2005-Tmax

Furthermore, we explore the biases produced by the validating datasets via bootstrapping approach. These biases are depicted by histograms in Fig. 9 and the mean presented in Table 5. Generally, the models used in prediction in PMA offer a better performance than Lisbon, with lower relative biases (See Table 5). The biases in winter for PMA is the lowest. All models biases exhibit less variability and also tend to cluster more tightly around zero, though there is much smaller variability in winter than summer and in LMA than in PMA. The results in Table 5 show in PMA, the negative bias in the number of deaths and positive bias for LMA prediction implying that validated models accuracies are less than the original data in Porto while the reverse is the case for Lisbon. However, as mentioned before, these differences are very small and centered around zero.

We carried out a parametric bootstrap simulation to study the influence of different choices of exposure-lag-response spline function on overall cumulative risk (Additional file 18: Table S2). Generally, all models have lower relative bias. The higher relative RMSE observed in Model 1–4 can be attributed to lack of fit, due to insufficient flexibility in the lag-response spline function. The model 11 specification has quadratic B-spline in both exposure-response and lag-response spline functions with 8 dfs for date to capture trend and seasonality. The coverage of the 95% CI from 500 simulations was 94.6%, bias of 1.9 and RMSE of 0.07 (Additional file 18: Table S2).

## Discussion

In the current study, we have developed different statistical models to assess the relationship between the circulatory disease mortality (CD) and temperatures (simulated and observed). Simulated temperature is previously subjected to bias correction.

The analysis was based on models fitted by a DLNM model to assess the effects of temperature on mortality [35, 37]. The DLNM was determined using only the first half of the data (1986–2000) and validated for the independent period of 2001–2005. The method was applied by using observed and simulated temperatures. We evaluated the error (RMSE and MAD) of the statistical method for the validation period. The main objective here is to validate the method using and independent period and also to validate the use of simulated temperatures which can be used for future climate scenarios.

Uncertainty is estimated by performing bootstrapping runs, over a 20-year period for Portuguese Metropolitan Areas (PMA, Porto Metropolitan Area; and LMA, Lisbon Metropolitan Area). Bootstrapping runs were performed to identify the best model candidate and to evaluate the influence of different choices of exposure-lag-response spline function on overall cumulative risk. To our best knowledge these have never been examined before in our country, using recent and consistent methods.

Results indicate that the associated risks for validated data are similar to the associated risks for training data. The estimated values of the RSME and MAD presented in our results are relatively small, implying a good prediction. The lower RSME provides evidence of accurate smoothing and thus good predictions based on the models. RSME and MAD show that the models provide a good prediction of the temperature effect on deaths from circulatory diseases, both in PML and in LMA. For Bias, considering the two seasons analyzed, the winter season is the one with the most underestimates, while summer is the season with highest overestimates. According the results, in LMA the Bias has a positive value for both Tmean and Tmin, which indicates that the forecast tend to overestimate the observed values. In PMA, for the two seasons, it is verified that in all the meteorological variables the Bias is negative. Our findings reported that after adjustment, forecasts tend to underestimate the observed values.

The risk of exposure was considered for lags between 0 and 30 days. Previous studies have suggested that short time lags cannot completely capture the effects of temperature on cardiovascular mortality [34]. Results indicate a non-linear association between exposure to temperature and mortality in both metropolitan areas being studied (PMA and LMA). The lagged association between mortality and temperature varies in the summer and winter in the study region. In both LMA and PMA, our findings indicated that there is an increase risk for 1–2 days lag (peak at lag 3) temperature-effect for CD due to extreme low daily mean temperatures (around 5 °C) in the winter months. However, strongest association in the summer months occur at 0-lag for extreme hot temperatures.

Results using both observed and simulated temperatures show an increased risk with extremely low mean temperatures in PMA during the cold season, gradually diminishing. Exposure to extreme daily minimum temperatures indicates lower risk, without being statistically significant. The highest risk is in the 1st percentile of minimum temperature. These results are in agreement with similar studies that found significant increased risk of death associated with cold temperatures [2, 11, 34]. During the warmer months, daily maximum/mean temperature effects in the summer are not significantly associated with mortality risks. Similar to Porto, in LMA, exposure-lag-response associations during the summer period indicate a lower associated risk of death, being non-significant with exposures to maximum temperatures in both periods. The risk is associated with average temperatures. During the cold months, exposure to daily minimum temperatures shows a higher associated risk of death compared to daily mean temperatures. The risk was highest for extreme minimum temperatures (1st percentile). Similar results were obtained in other studies carried out in countries such as the Portugal [11] and USA [38, 39], reporting stronger associations between extremely low temperatures and circulatory disease mortality. Overall, results obtained using simulated temperature are similar or even better than those using observed temperature.

Moreover, we applied in this study a bootstrap approach to estimate the uncertainty of the DLNM model to the validating datasets. These indicate that the predictions for PMA have a better performance than those for LMA. During the cold season, they are lower in Porto. In all the models, biases have a lower variability in LMA compared to Porto and are close to zero. Our results show that, despite the aforesaid differences between both metropolitan areas, these are minimal and close to zero. This gives an indication of the robustness of the methods [18] and both methods are able to reduce biases in the models fields significantly.

A key issue in multi-parameter study and DLNM is the selection of appropriate spline functions to capture the exposure-lag-response relationship. In this study, we used QAIC to select the best fitting model under different scenarios. We were able to properly compare the overall cumulative effect estimated under different bi-dimensional exposure-lag-response relationship and described the uncertainty in the estimate. Our parametric bootstrap simulation study suggested that the estimated overall cumulative effects are relatively stable especially for model selected by QAIC. Previous studies have reported that the overfitting characterized by AIC-selected models in different simple exposure-lag-response dependencies does not seriously affect its performance [23, 40].

As previously mentioned, we observed regional differences regarding the estimated risk for each of the metropolitan areas under analysis. Applying alert systems implemented by the Portuguese Directorate-General of Health [12, 13] has played an important role in preventing deaths and diseases, but the early warning system is not efficient on its own, since it cannot offer an effective prediction in case of short-, medium-, and long-term extreme events. Early warning systems [41, 42], months or years ahead of the actual occurrence of extreme events, may give health authorities and urban planners enough time to update climate adaptation plans [43], effective adaptation and mitigation plans at local level, in the short-, medium-, and long-term. This may be of great importance to public health prevention, because cities and metropolitan areas are vulnerable to climate change due to their population and infrastructure density [44]. Climate models clearly show that the Mediterranean region is one of the areas most influenced by current and future climate change [45]. The climate change is increasing the frequency, intensity, duration of heat waves in general [46–48] and increased population life expectancies imply that the health protection of elderly people will become a major challenge for all Mediterranean countries. [45, 49]. In this context, in Lisbon and Porto metropolitan areas monitoring and predicted climate change health impacts and adaptation measures should be a priority for health care planning. Similar to Portugal, several countries, regions, and cities still do not have such action plans in place [44, 50].

## Conclusions

The aim of this study is to develop a method to identify the relationship between extreme temperatures and mortality risk by using as predictors simulated temperature data for cold and hot conditions in two urban areas in Portugal. For that, simulated temperature for cold and hot conditions in PMA and LMA, for 1986–2000 and 2001–2005 were used. Results of RR using observed and simulated temperatures are similar and even better for the latter. The developed DLNM model was determined for the 1986–2000 period and was validated in the independent period of 2001–2005. Bootstrapping runs were performed to evaluate the uncertainty of the model. The use of QAIC to choose from several candidate models has shown to be a reasonable approach in reducing uncertainty in the estimated RR for circulatory diseases mortality. A good agreement is found for PMA. The statistical evaluation parameters presented confirm that the simulation for summer is the one with better results, due to the lower RMSE, MAD and Bias values in temperature. The lower RSME provide evidence of accurate smoothing and thus prediction based on the models. Prediction error is lower for these DLNM models, indicated by the closeness between the fitted training set (1986–2000) and prediction test set (2001–2005). Considering these results, the first fact that becomes clear is that the model is somewhat sensitive to whether it is simulating cold periods.

In our country, not much is known about developing predictive models with the aim of creating early warning systems, health prevention plans and local climate action plans. We believe adequate communication strategies and timely response capabilities would be more effective if climate models were implemented and included (on a global or regional scale) in the prevention programs established in Portugal by health authorities. Climate prediction would allow a better resource management in the short-, medium-, and long-term in warning systems nationally, and with primary healthcare providers a local level.

## Additional files

Additional file 1: **Table S1.** Model parameter selection for different exposure-response and lag-response functions and degrees of freedom (df) to capture trend and seasonality. (DOCX 16 kb)

Additional file 2: **Figure S1.** Exposure-lag-response, overall cumulative exposure-response, and lag-response for observed data in WINTER for Porto Metropolitan Area (PMA), during 1986–2000 using minimum temperature (Tmin). Figure S1 presents mortality-exposure association in PMA (Winter 1986–2000), however using daily minimum temperature exposure. The exposure–lag–response show an initial increase in RR along lags, peaking at approximately 3 days after cumulative exposure minimum temperature of − 0.3 °C (1st percentile). Exposure to extreme minimum daily temperatures (less the − 1 °C) indicates lower RR and were not statistically significant. (TIF 327 kb)

Additional file 3: **Figure S2.** Exposure-lag-response, overall cumulative exposure - response, and lag-response for observed data in SUMMER for Porto Metropolitan Area (PMA), during 1986–2000 using maximum temperature (Tmax). (TIF 333 kb)

Additional file 4: **Figure S3.** Exposure -lag-response, overall cumulative exposure - response, and lag-response for observed data in WINTER for Lisbon Metropolitan Area (LMA), during 1986–2000 using minimum temperature (Tmin). (TIF 344 kb)

Additional file 5: **Figure S4.** Exposure -lag-response, overall cumulative exposure - response, and lag-response for observed data in SUMMER for Lisbon Metropolitan Area (LMA), during 1986–2000 using maximum temperature (Tmax). (TIF 325 kb)

Additional file 6: **Figure S5.** Exposure -lag-response, overall cumulative exposure - response, and lag-response for **simulated** data in SUMMER for Lisbon Metropolitan Area (LMA), during 1986–2000 using maximum temperature (Tmax). (TIF 329 kb)

Additional file 7: **Figure S6.** Exposure -lag-response, overall cumulative exposure - response, and lag-response for **simulated** data in WINTER for Lisbon Metropolitan Area (LMA), during 1986–2000 using minimum temperature (Tmin). (TIF 307 kb)

Additional file 8: **Figure S7.** Exposure -lag-response, overall cumulative exposure - response, and lag-response for **simulated** data in SUMMER for Porto Metropolitan Area (PMA), during 1986–2000 using maximum temperature (Tmax). (TIF 303 kb)

Additional file 9: **Figure S8.** Exposure -lag-response, overall cumulative exposure - response, and lag-response for **simulated** data in WINTER for Porto Metropolitan Area (PMA), during 1986–2000 using minimum temperature (Tmin). (TIF 339 kb)

Additional file 10: **Figure S9.** Predictive assessment of mortality in PMA, 1986–2000 (Winter) using 2001–2005 data. DLNM with (a) mean temperature (Tmean), (b) minimum temperature (Tmin). The left panel is the histogram of residuals comparing the risk association prediction for 1986–2000 and 2001–2005, using: (a) mean temperature (Tmean) and (b) minimum temperature (Tmin). The right panel presents the overall cumulative exposure-response association with the red solid line representing the estimates from 1986 to 2000, while the blue dashed line represents the estimates from the 2001–2005. (TIF 290 kb)

Additional file 11: **Figure S10.** Predictive assessment of mortality in PMA, 1986–2000 (Summer) using 2001–2005 data. DLNM with (a) mean temperature (Tmean), (b) maximum temperature (Tmax). The left panel is the histogram of residuals comparing the risk association prediction for 1986–2000 and 2001–2005, using: (a) mean temperature (Tmean) and (b) maximum temperature (Tmax). The right panel presents the overall cumulative exposure-response association with the red solid line representing the estimates from 1986 to 2000, while the blue dashed line represents the estimates from the 2001–2005. (TIF 278 kb)

Additional file 12: **Figure S11.** Predictive assessment of mortality in LMA, 1986–2000 (Winter) using 2001–2005 data. DLNM with (a) mean temperature (Tmean), (b) minimum temperature (Tmin). The left panel is the histogram of residuals comparing the risk association prediction for 1986–2000 and 2001–2005, using: (a) mean temperature (Tmean) and (b) minimum temperature (Tmin). The right panel presents the overall cumulative exposure-response association with the red solid line representing the estimates from 1986 to 2000, while the blue dashed line represents the estimates from the 2001–2005. (TIF 244 kb)

Additional file 13: Figure S12 Predictive assessment of mortality in LMA, 1986–2000 (Summer) using 2001–2005 data. DLNM with (a) mean temperature (Tmean), (b) maximum temperature (Tmax). The left panel is the histogram of residuals comparing the risk association prediction for 1986–2000 and 2001–2005, using: (a) mean temperature (Tmean) and (b) maximum temperature (Tmax). The right panel presents the overall cumulative exposure-response association with the red solid line representing the estimates from 1986 to 2000, while the blue dashed line represents the estimates from the 2001–2005. (TIF 287 kb)

Additional file 14: **Figure S13.** Predictive mortality assessment for simulated data for LMA, 1986–2000 (summer) using 2001–2005 data. DLNM with (a) mean temperature (Tmean), (b) maximum temperature (Tmax). The left panel is the histogram of residuals comparing the risk association prediction for 1986–2000 and 2001–2005, using: (a) mean temperature (Tmean) and (b) maximum temperature (Tmax). The right panel presents the overall cumulative exposure-response association with the red solid line representing the estimates from 1986 to 2000, while the blue dashed line represents the estimates from the 2001–2005. (TIF 266 kb)

Additional file 15: **Figure S14.** Predictive mortality assessment for simulated data for LMA, 1986–2000 (winter) using 2001–2005 data. DLNM with (a) mean temperature (Tmean), (b) minimum temperature (Tmin). The left panel is the histogram of residuals comparing the risk association prediction for 1986–2000 and 2001–2005, using: (a) mean temperature (Tmean) and (b) minimum temperature (Tmin). The right panel presents the overall cumulative exposure-response association with the red solid line representing the estimates from 1986 to 2000, while the blue dashed line represents the estimates from the 2001–2005. (TIF 287 kb)

Additional file 16: **Figure S15.** Predictive mortality assessment for simulated data for PMA, 1986–2000 (summer) using 2001–2005 data. DLNM with (a) mean temperature (Tmean), (b) maximum temperature (Tmax). The left panel is the histogram of residuals comparing the risk association prediction for 1986–2000 and 2001–2005, using: (a) mean temperature (Tmean) and (b) maximum temperature (Tmax). The right panel presents the overall cumulative exposure-response association with the red solid line representing the estimates from 1986 to 2000, while the blue dashed line represents the estimates from the 2001–2005. (TIF 271 kb)

Additional file 17: **Figure S16.** Predictive mortality assessment for simulated data for PMA, 1986–2000 (summer) using 2001–2005 data. DLNM with (a) mean temperature (Tmean), (b) minimum temperature (Tmin). The left panel is the histogram of residuals comparing the risk association prediction for 1986–2000 and 2001–2005, using: (a) mean temperature (Tmean) and (b) minimum temperature (Tmin). The right panel presents the overall cumulative exposure-response association with the red solid line representing the estimates from 1986 to 2000, while the blue dashed line represents the estimates from the 2001–2005. (TIF 297 kb)

Additional file 18: **Table S2.** Summaries of synthetic indices of relative bias, coverage and relative root mean square error (RMSE) for different parameterization of exposure-lag-response spline function with 8df for DATE to capture trend and seasonality. (DOCX 14 kb)

## Abbreviations

CD: Circulatory disease mortality
CI: Confidence Interval
DLNM: Distributed lag nonlinear model
LMA: Lisbon Metropolitan Area
MAD: Mean absolute deviation
MPI-ESM-LR: Max Planck Institute for Meteorology Earth System Model
PMA: Porto Metropolitan Area
QAIC: Quasi-Akaike Information Criteria
RMSE: Root mean square error
RR: Relative risk
WRF: Weather Research and Forecast
