# Supplementary material for: Bootstrap approach to validate the performance of models for predicting mortality risk temperature in Portuguese Metropolitan Areas
Source: Environ Health. 2019 Mar 29;18:25. doi: 10.1186/s12940-019-0462-x (PMC6440075; doi:10.1186/s12940-019-0462-x)
Supplement: Supplementary file 1 — Table S1. Model parameter selection for different exposure-response and lag-response functions and degrees of freedom (df) to capture trend and seasonality. (DOCX 16 kb) [file 12940_2019_462_MOESM1_ESM.docx]

**Table S1** Model parameter selection for different exposure-response and lag-response functions and degrees of freedom (df) to capture trend and seasonality.

| **Model** | **Exposure-response** | **Lag-response** |  | **Q-AIC** | | |
| --- | --- | --- | --- | --- | --- | --- |
|  |  |  | **Total df** | **Date, df=7** | **Date, df=8** | **Date, df=9** |
| Model 1 | linear | Constant | 1 | 12872.4 | 12851.4 | 12866.6 |
| Model 2 | Quadratic B-Spline^a^ | Constant | 2 | 12873.1 | 12852.8 | 12867.8 |
| Model 3 | Quadratic B-Spline^b^ | Constant | 3 | 12872.6 | 12852.5 | 12867.1 |
| Model 4 | Quadratic B-Spline^c^ | Constant | 4 | 12879.3 | 12859.9 | 12874.3 |
|  |  |  |  |  |  |  |
| Model 5 | linear | Quadratic B-Spline^d^ | 3 | 12846.9 | 12827.7 | 12841.9 |
| Model 6 | Quadratic B-Spline^a^ | Quadratic B-Spline^d^ | 6 | 12790.3 | 12770 | 12784.2 |
| Model 7 | Quadratic B-Spline^b^ | Quadratic B-Spline^d^ | 9 | 12790.3 | 12773.3 | 12785.2 |
| Model 8 | Quadratic B-Spline^c^ | Quadratic B-Spline^d^ | 12 | 12790.3 | 12777.4 | 12786.7 |
|  |  |  |  |  |  |  |
| Model 9 | linear | Quadratic B-Spline^e^ | 4 | 12839.3 | 12820.9 | 12834.5 |
| Model 10 | Quadratic B-Spline^a^ | Quadratic B-Spline^e^ | 18 | 12785.1 | 12767.4 | 12779.2 |
| Model 11 | Quadratic B-Spline^b^ | Quadratic B-Spline^e^ | 12 | 12781.8 | **12765.2** | 12776.8 |
| Model 12 | Quadratic B-Spline^c^ | Quadratic B-Spline^e^ | 16 | 12782.5 | 12769.5 | 12779.2 |
|  |  |  |  |  |  |  |
| Model 13 | linear | Quadratic B-Spline^f^ | 5 | 12842.4 | 12824.3 | 12837.7 |
| Model 14 | Quadratic B-Spline^a^ | Quadratic B-Spline^f^ | 10 | 12787.2 | 12769.9 | 12781.6 |
| Model 15 | Quadratic B-Spline^b^ | Quadratic B-Spline^f^ | 15 | 12788.9 | 12772.4 | 12784 |
| Model 16 | Quadratic B-Spline^c^ | Quadratic B-Spline^f^ | 20 | 12793.5 | 12780.6 | 12790.3 |
