# Supplementary figures and images for: Bootstrap approach to validate the performance of models for predicting mortality risk temperature in Portuguese Metropolitan Areas
Source: Environ Health. 2019 Mar 29;18:25. doi: 10.1186/s12940-019-0462-x (PMC6440075; doi:10.1186/s12940-019-0462-x)

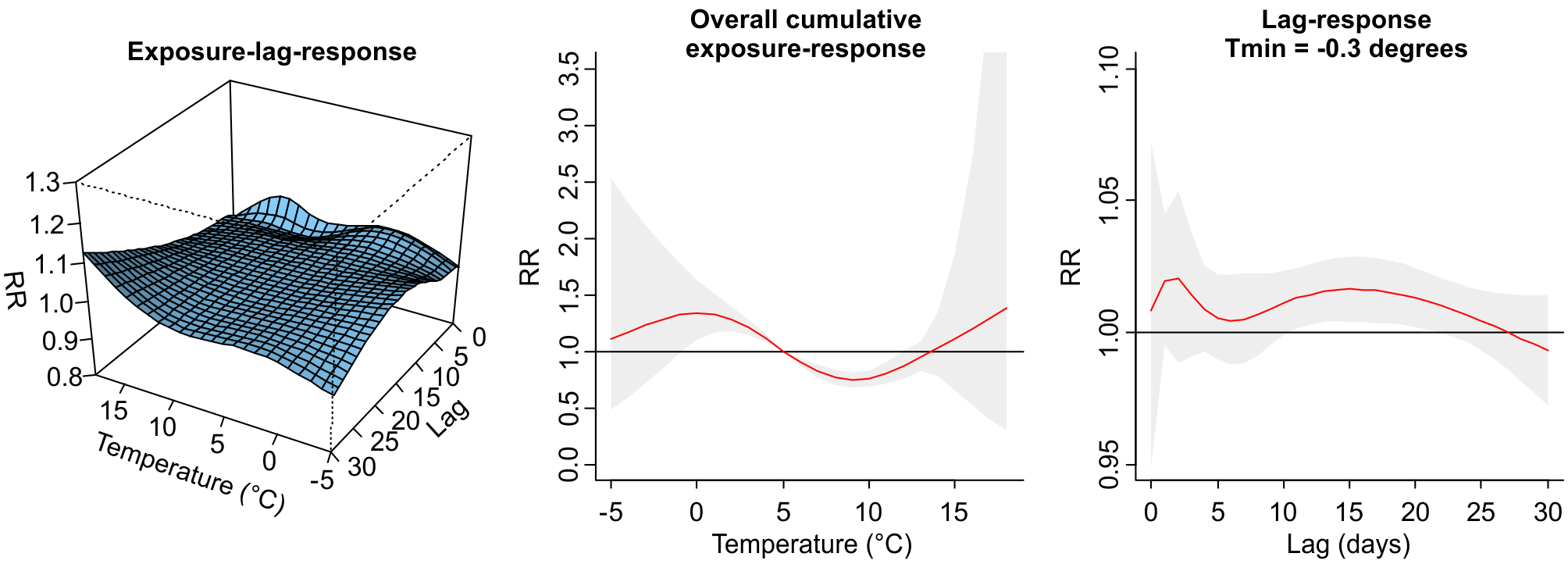

Supplement: Supplementary file 2 — Figure S1. Exposure-lag-response, overall cumulative exposure-response, and lag-response for observed data in WINTER for Porto Metropolitan Area (PMA), during 1986–2000 using minimum temperature (Tmin). Figure S1 presents mortality-exposure association in PMA (Winter 1986–2000), however using daily minimum temperature exposure. The exposure–lag–response show an initial increase in RR along lags, peaking at approximately 3 days after cumulative exposure minimum temperature of − 0.3 °C (1st percentile). Exposure to extreme minimum daily temperatures (less the − 1 °C) indicates lower RR and were not statistically significant. (TIF 327 kb) [file 12940_2019_462_MOESM2_ESM.tif]

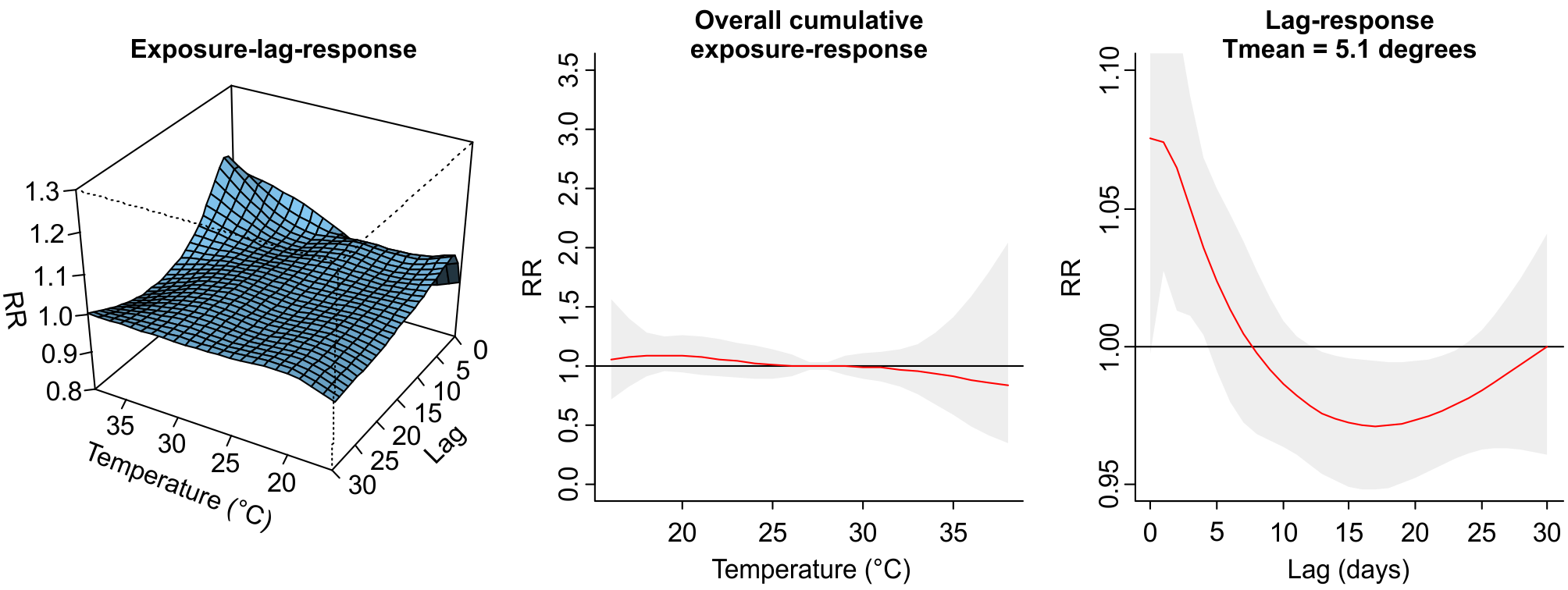

Supplement: Supplementary file 3 — Figure S2. Exposure-lag-response, overall cumulative exposure - response, and lag-response for observed data in SUMMER for Porto Metropolitan Area (PMA), during 1986–2000 using maximum temperature (Tmax). (TIF 333 kb) [file 12940_2019_462_MOESM3_ESM.tif]

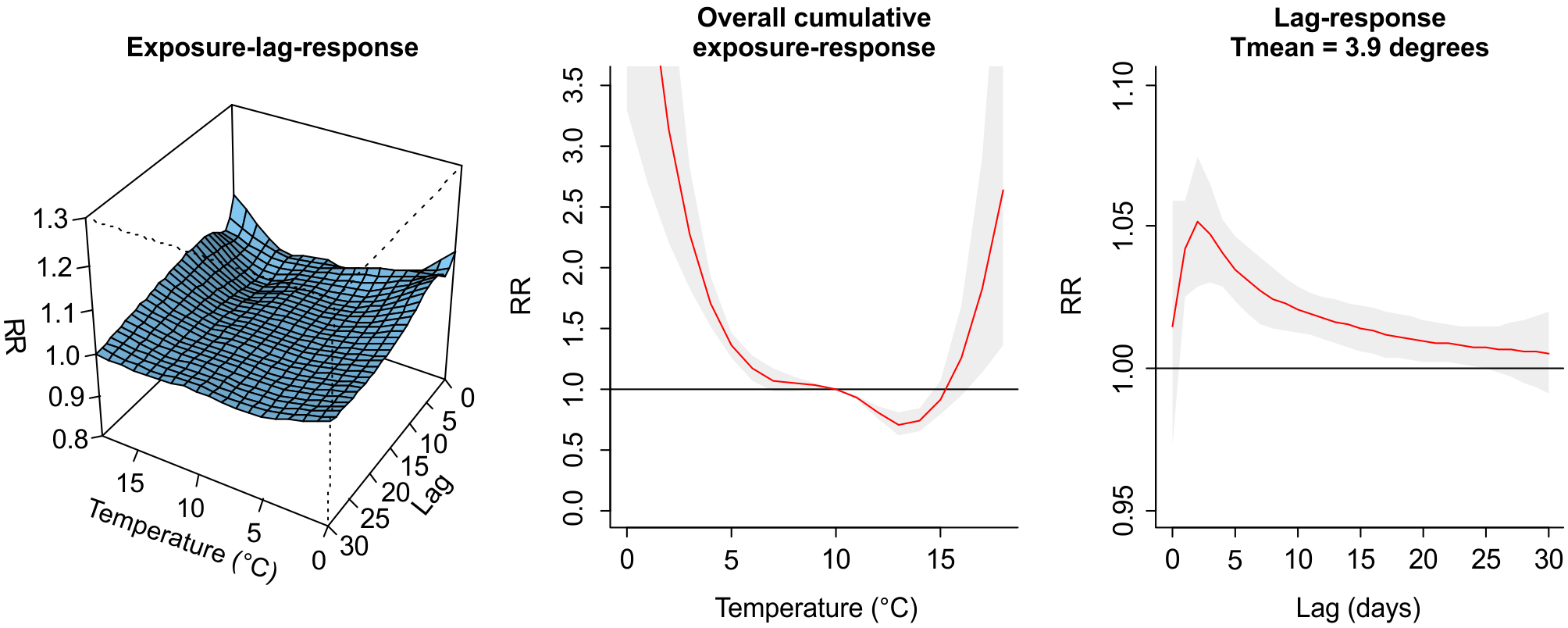

Supplement: Supplementary file 4 — Figure S3. Exposure -lag-response, overall cumulative exposure - response, and lag-response for observed data in WINTER for Lisbon Metropolitan Area (LMA), during 1986–2000 using minimum temperature (Tmin). (TIF 344 kb) [file 12940_2019_462_MOESM4_ESM.tif]

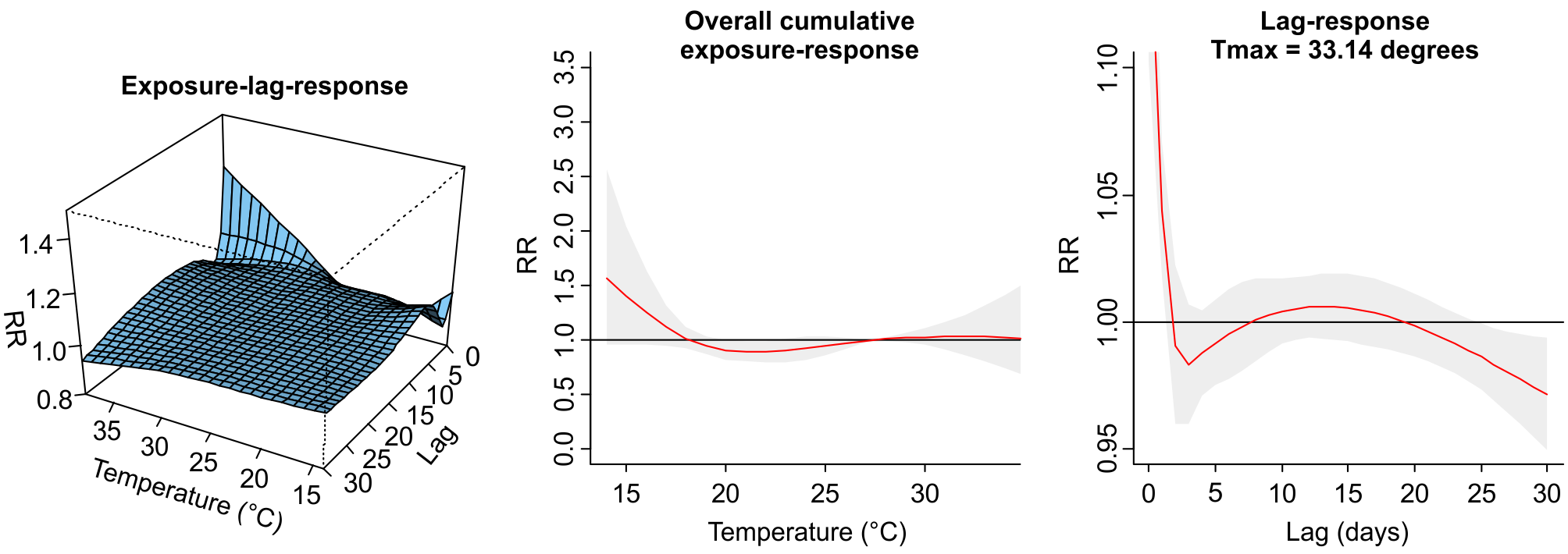

Supplement: Supplementary file 5 — Figure S4. Exposure -lag-response, overall cumulative exposure - response, and lag-response for observed data in SUMMER for Lisbon Metropolitan Area (LMA), during 1986–2000 using maximum temperature (Tmax). (TIF 325 kb) [file 12940_2019_462_MOESM5_ESM.tif]

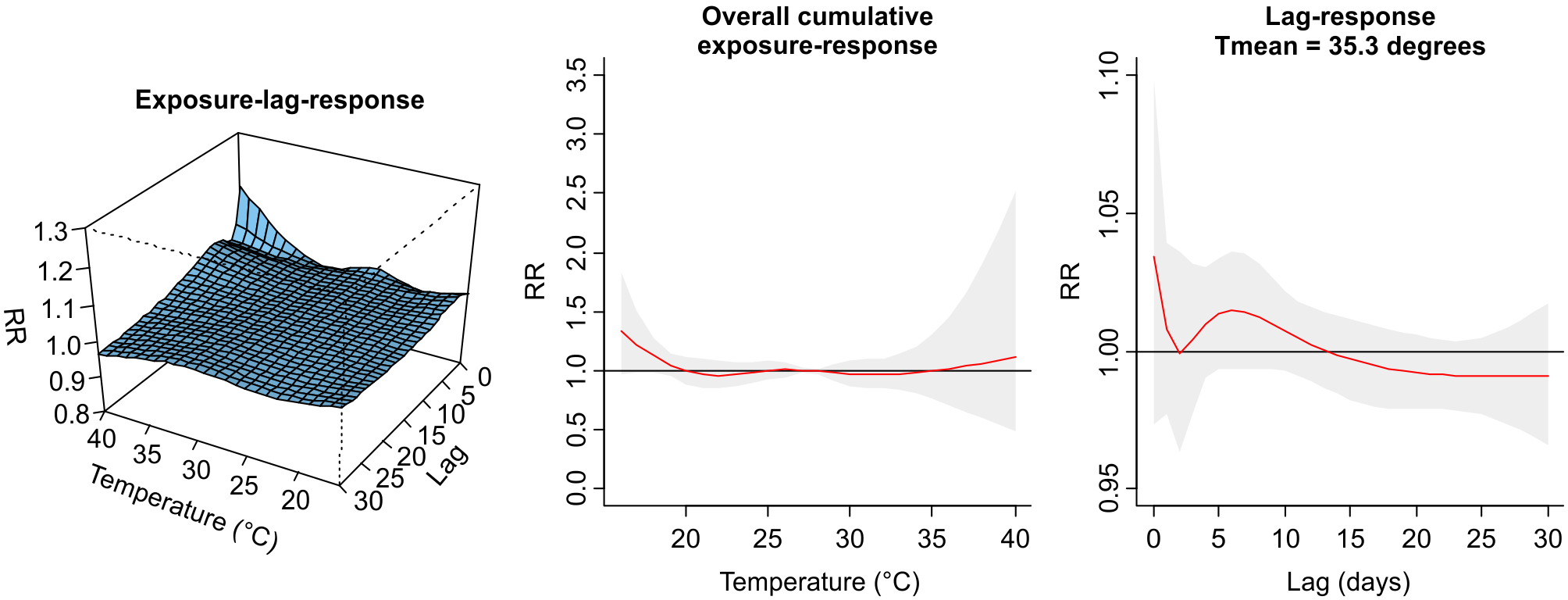

Supplement: Supplementary file 6 — Figure S5. Exposure -lag-response, overall cumulative exposure - response, and lag-response for simulated data in SUMMER for Lisbon Metropolitan Area (LMA), during 1986–2000 using maximum temperature (Tmax). (TIF 329 kb) [file 12940_2019_462_MOESM6_ESM.tif]

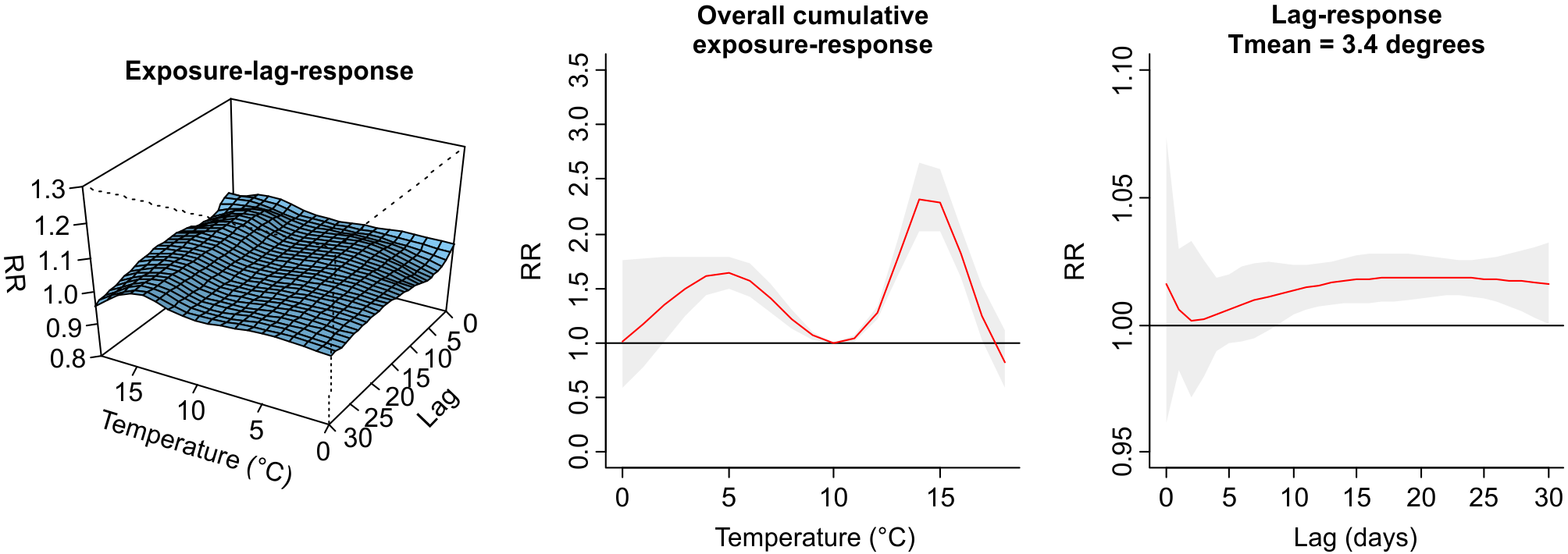

Supplement: Supplementary file 7 — Figure S6. Exposure -lag-response, overall cumulative exposure - response, and lag-response for simulated data in WINTER for Lisbon Metropolitan Area (LMA), during 1986–2000 using minimum temperature (Tmin). (TIF 307 kb) [file 12940_2019_462_MOESM7_ESM.tif]

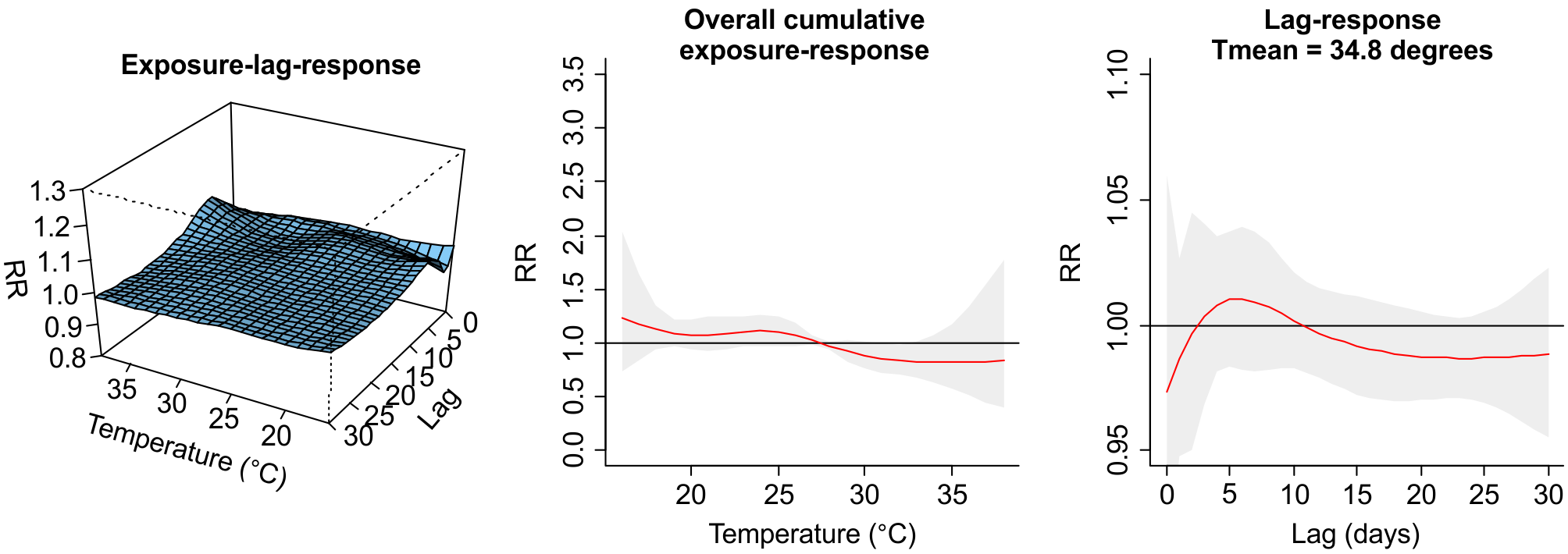

Supplement: Supplementary file 8 — Figure S7. Exposure -lag-response, overall cumulative exposure - response, and lag-response for simulated data in SUMMER for Porto Metropolitan Area (PMA), during 1986–2000 using maximum temperature (Tmax). (TIF 303 kb) [file 12940_2019_462_MOESM8_ESM.tif]

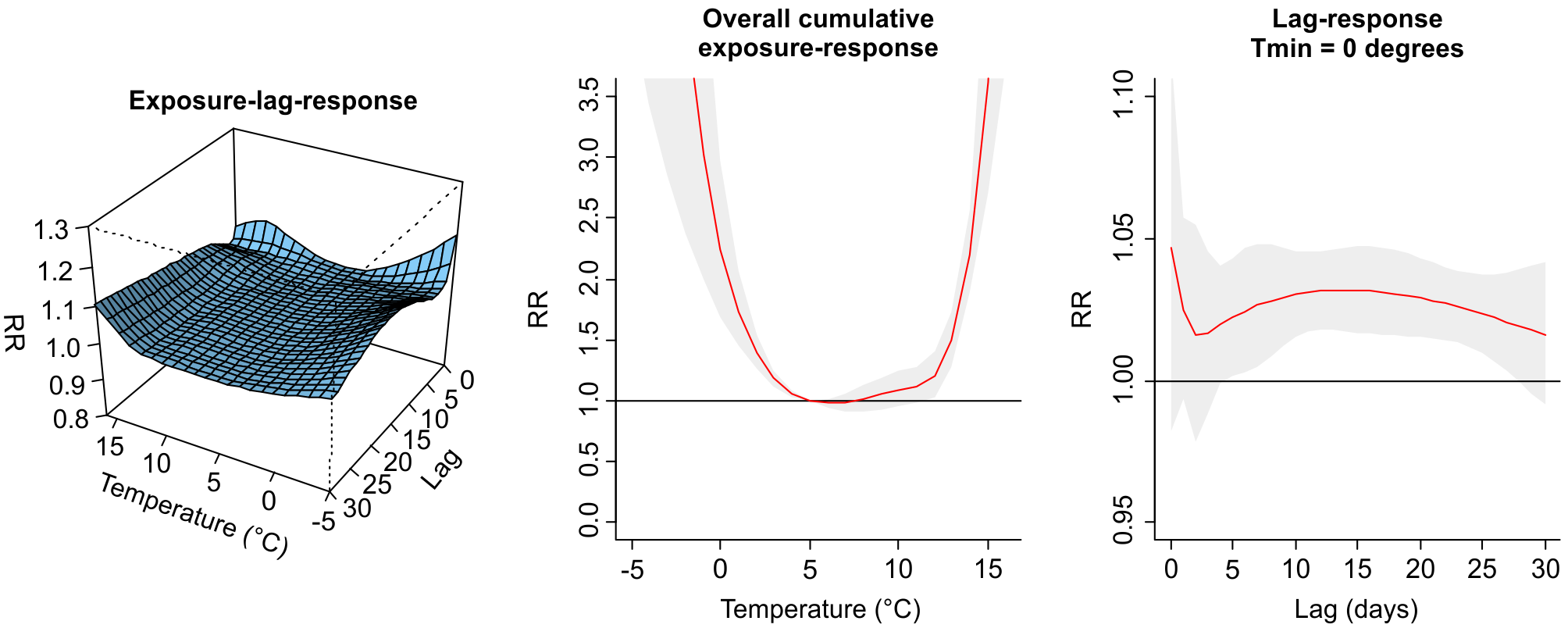

Supplement: Supplementary file 9 — Figure S8. Exposure -lag-response, overall cumulative exposure - response, and lag-response for simulated data in WINTER for Porto Metropolitan Area (PMA), during 1986–2000 using minimum temperature (Tmin). (TIF 339 kb) [file 12940_2019_462_MOESM9_ESM.tif]

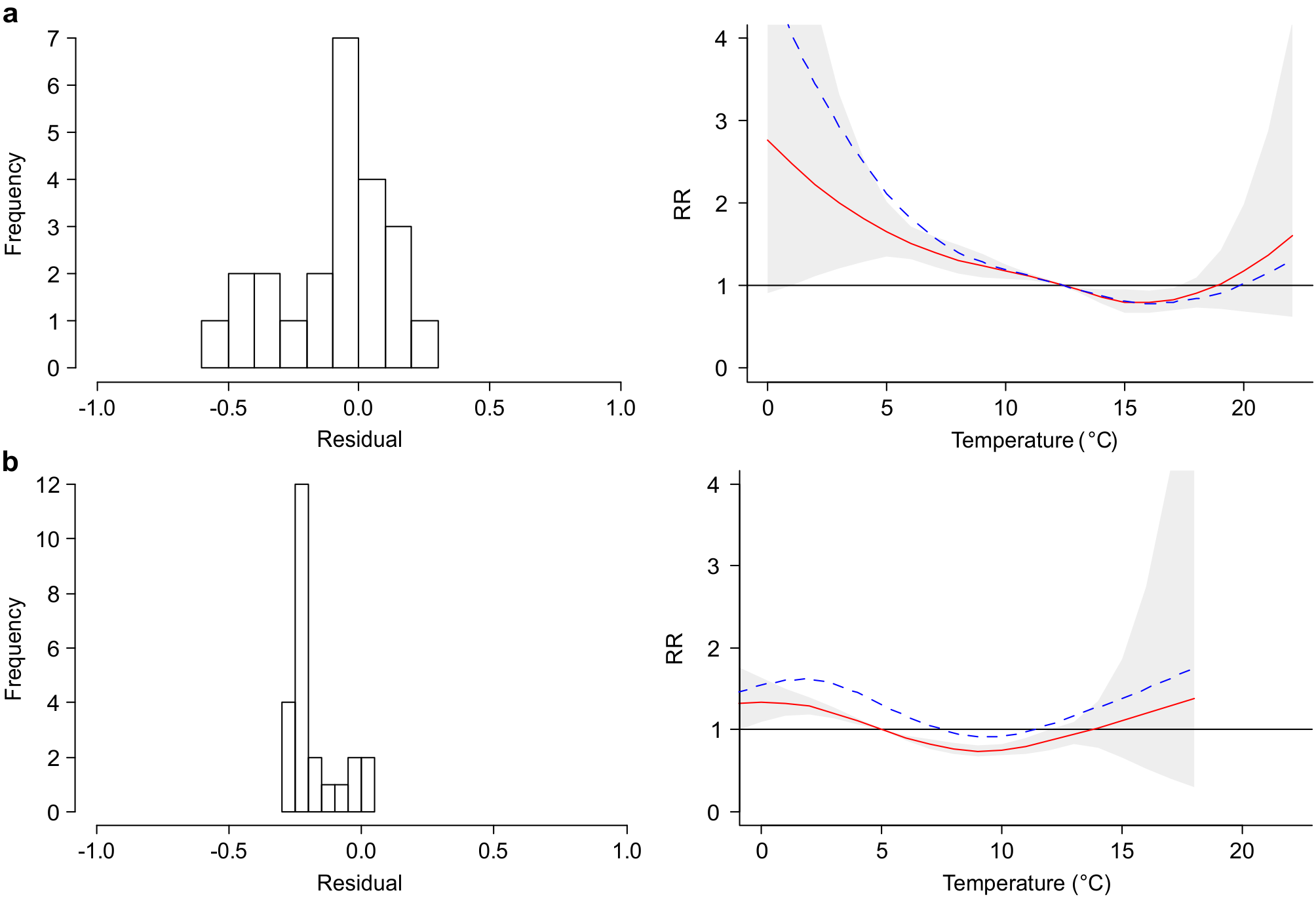

Supplement: Supplementary file 10 — Figure S9. Predictive assessment of mortality in PMA, 1986–2000 (Winter) using 2001–2005 data. DLNM with (a) mean temperature (Tmean), (b) minimum temperature (Tmin). The left panel is the histogram of residuals comparing the risk association prediction for 1986–2000 and 2001–2005, using: (a) mean temperature (Tmean) and (b) minimum temperature (Tmin). The right panel presents the overall cumulative exposure-response association with the red solid line representing the estimates from 1986 to 2000, while the blue dashed line represents the estimates from the 2001–2005. (TIF 290 kb) [file 12940_2019_462_MOESM10_ESM.tif]

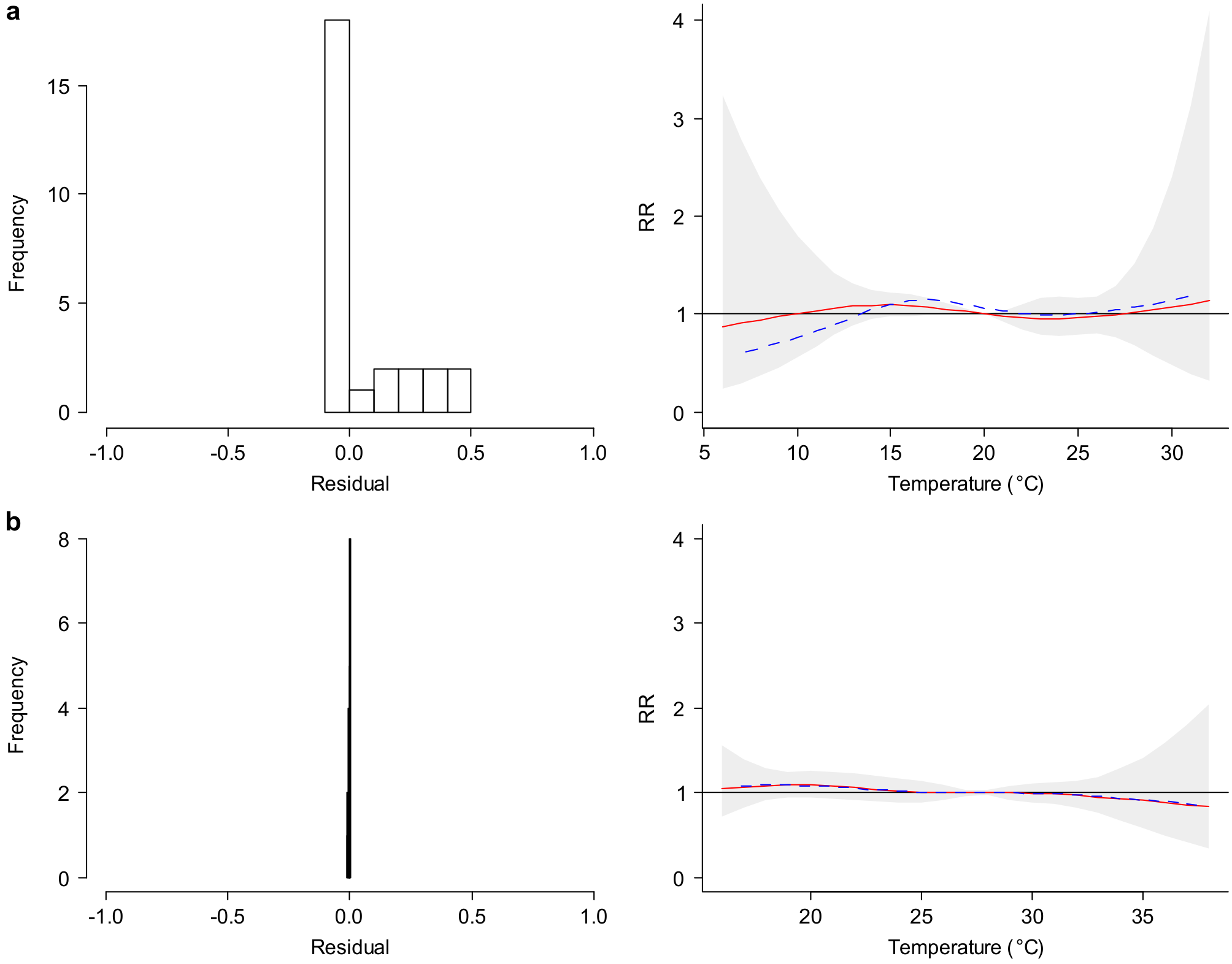

Supplement: Supplementary file 11 — Figure S10. Predictive assessment of mortality in PMA, 1986–2000 (Summer) using 2001–2005 data. DLNM with (a) mean temperature (Tmean), (b) maximum temperature (Tmax). The left panel is the histogram of residuals comparing the risk association prediction for 1986–2000 and 2001–2005, using: (a) mean temperature (Tmean) and (b) maximum temperature (Tmax). The right panel presents the overall cumulative exposure-response association with the red solid line representing the estimates from 1986 to 2000, while the blue dashed line represents the estimates from the 2001–2005. (TIF 278 kb) [file 12940_2019_462_MOESM11_ESM.tif]

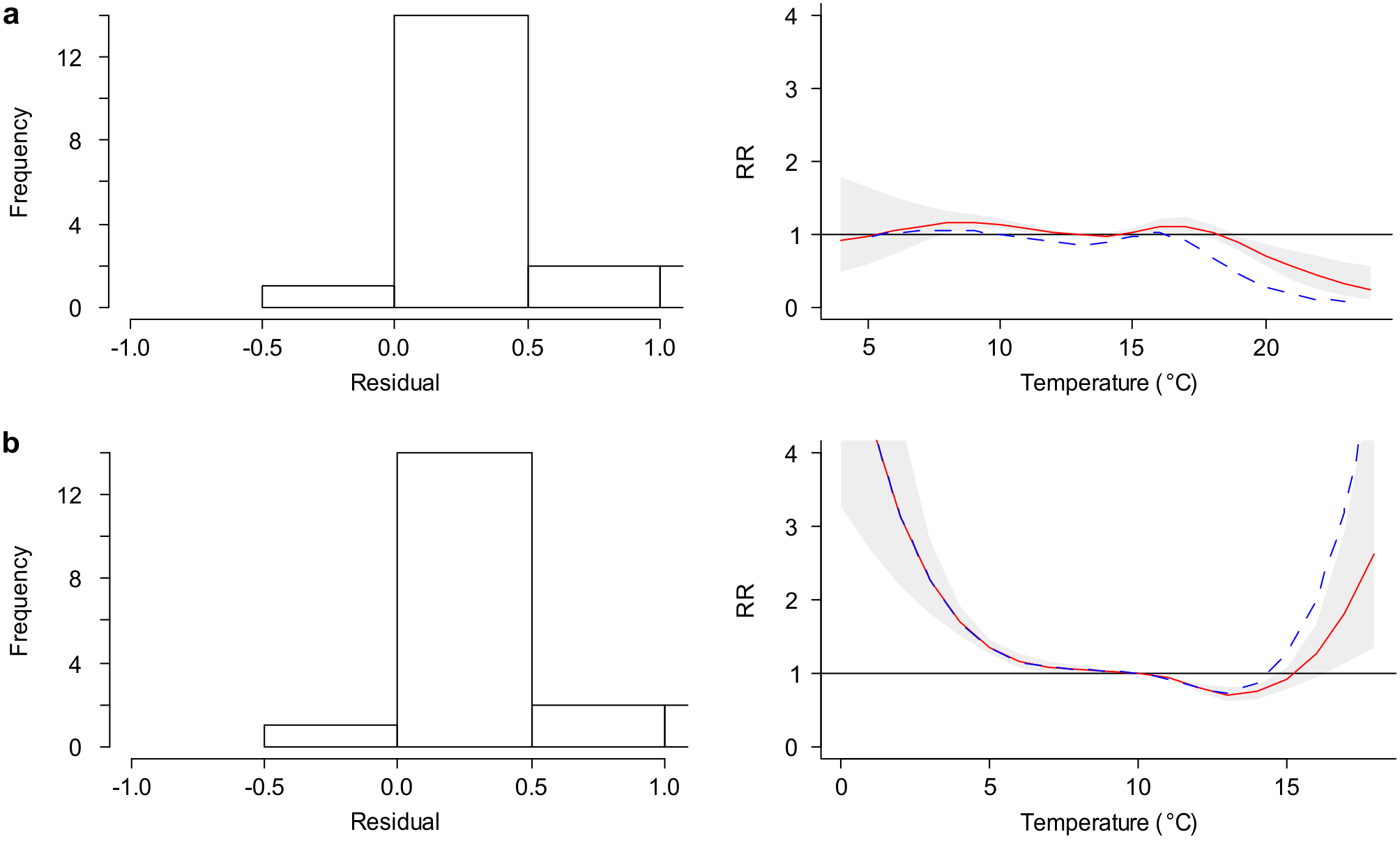

Supplement: Supplementary file 12 — Figure S11. Predictive assessment of mortality in LMA, 1986–2000 (Winter) using 2001–2005 data. DLNM with (a) mean temperature (Tmean), (b) minimum temperature (Tmin). The left panel is the histogram of residuals comparing the risk association prediction for 1986–2000 and 2001–2005, using: (a) mean temperature (Tmean) and (b) minimum temperature (Tmin). The right panel presents the overall cumulative exposure-response association with the red solid line representing the estimates from 1986 to 2000, while the blue dashed line represents the estimates from the 2001–2005. (TIF 244 kb) [file 12940_2019_462_MOESM12_ESM.tif]

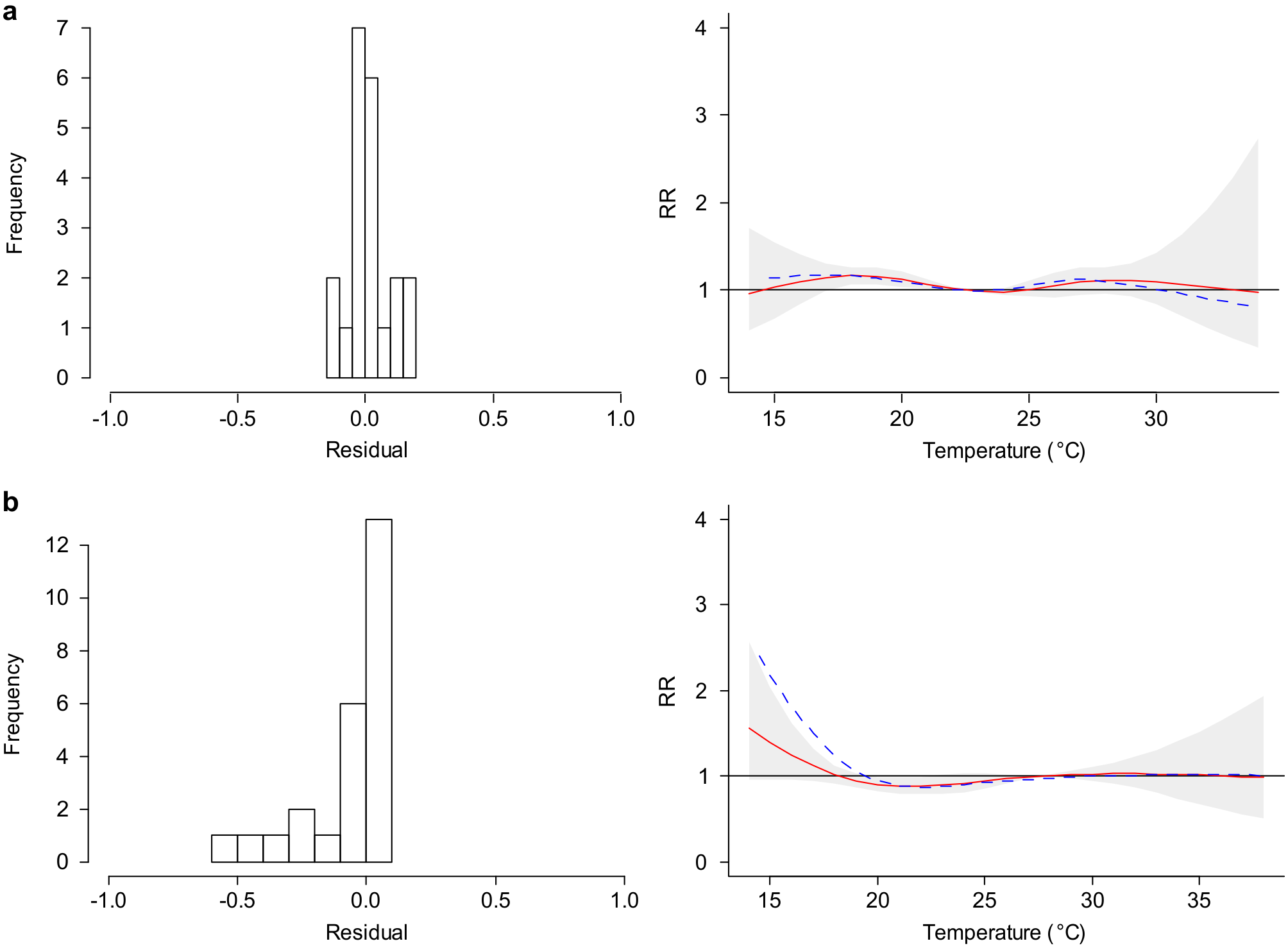

Supplement: Supplementary file 13 — Figure S12 Predictive assessment of mortality in LMA, 1986–2000 (Summer) using 2001–2005 data. DLNM with (a) mean temperature (Tmean), (b) maximum temperature (Tmax). The left panel is the histogram of residuals comparing the risk association prediction for 1986–2000 and 2001–2005, using: (a) mean temperature (Tmean) and (b) maximum temperature (Tmax). The right panel presents the overall cumulative exposure-response association with the red solid line representing the estimates from 1986 to 2000, while the blue dashed line represents the estimates from the 2001–2005. (TIF 287 kb) [file 12940_2019_462_MOESM13_ESM.tif]

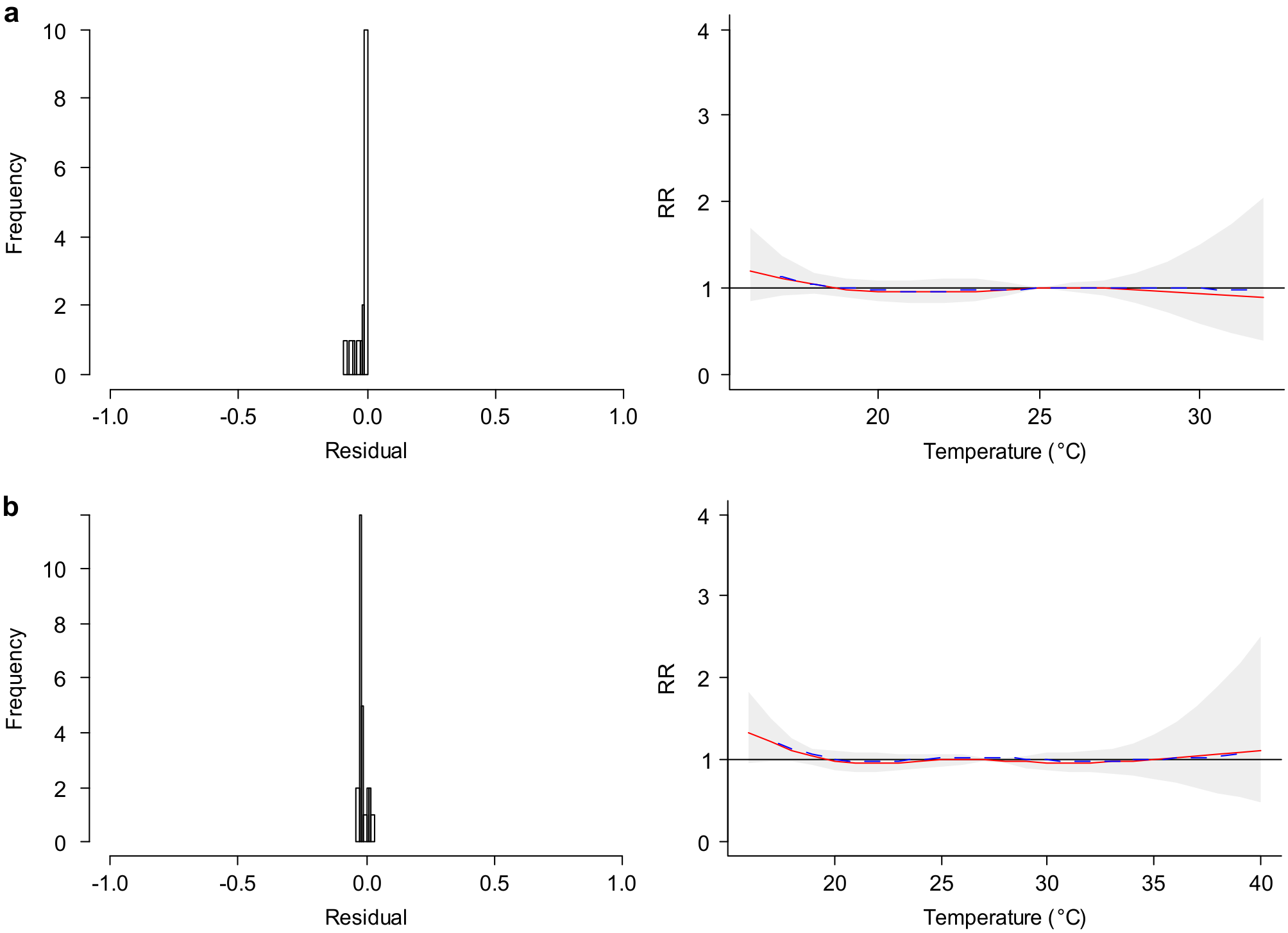

Supplement: Supplementary file 14 — Figure S13. Predictive mortality assessment for simulated data for LMA, 1986–2000 (summer) using 2001–2005 data. DLNM with (a) mean temperature (Tmean), (b) maximum temperature (Tmax). The left panel is the histogram of residuals comparing the risk association prediction for 1986–2000 and 2001–2005, using: (a) mean temperature (Tmean) and (b) maximum temperature (Tmax). The right panel presents the overall cumulative exposure-response association with the red solid line representing the estimates from 1986 to 2000, while the blue dashed line represents the estimates from the 2001–2005. (TIF 266 kb) [file 12940_2019_462_MOESM14_ESM.tif]

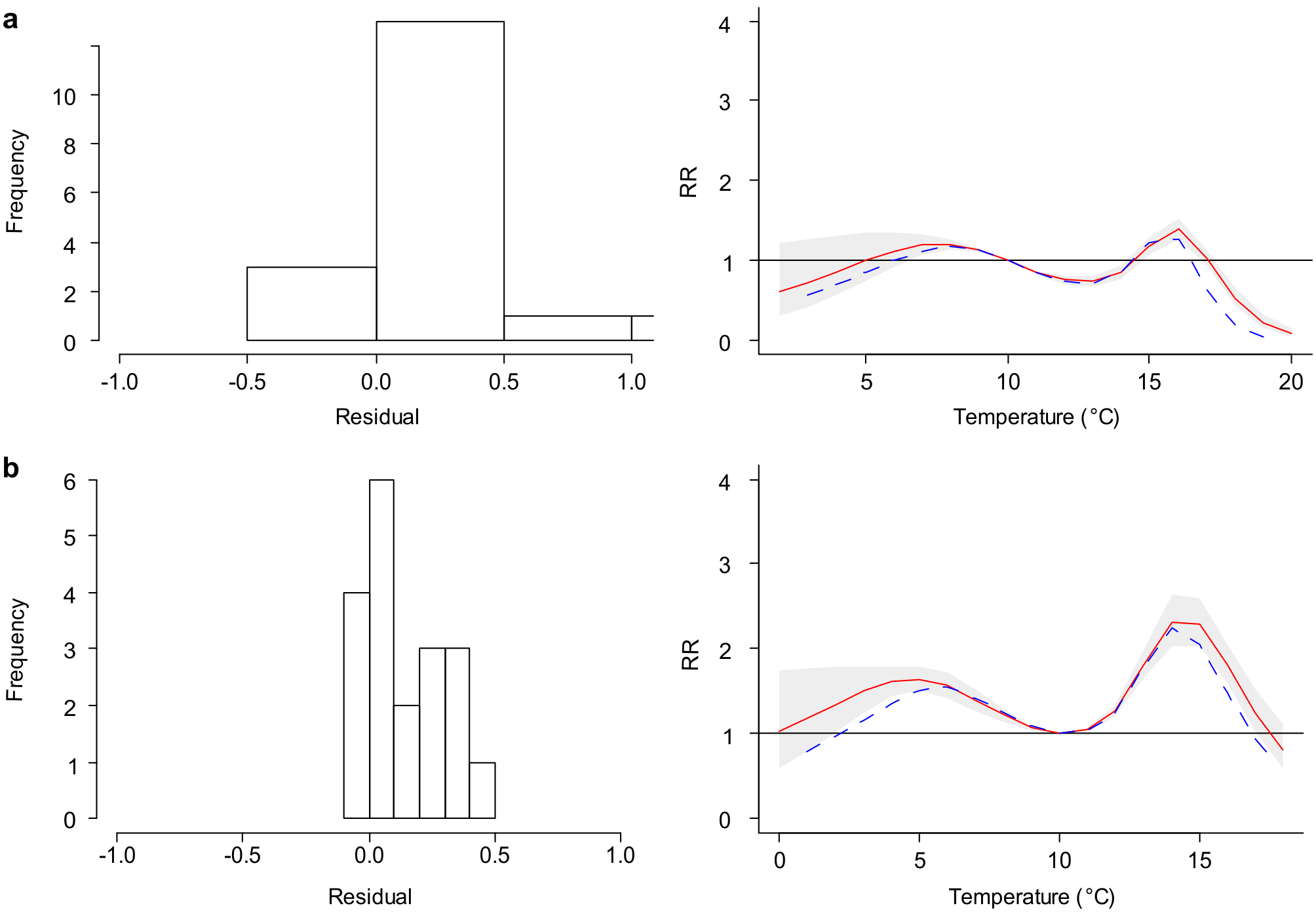

Supplement: Supplementary file 15 — Figure S14. Predictive mortality assessment for simulated data for LMA, 1986–2000 (winter) using 2001–2005 data. DLNM with (a) mean temperature (Tmean), (b) minimum temperature (Tmin). The left panel is the histogram of residuals comparing the risk association prediction for 1986–2000 and 2001–2005, using: (a) mean temperature (Tmean) and (b) minimum temperature (Tmin). The right panel presents the overall cumulative exposure-response association with the red solid line representing the estimates from 1986 to 2000, while the blue dashed line represents the estimates from the 2001–2005. (TIF 287 kb) [file 12940_2019_462_MOESM15_ESM.tif]

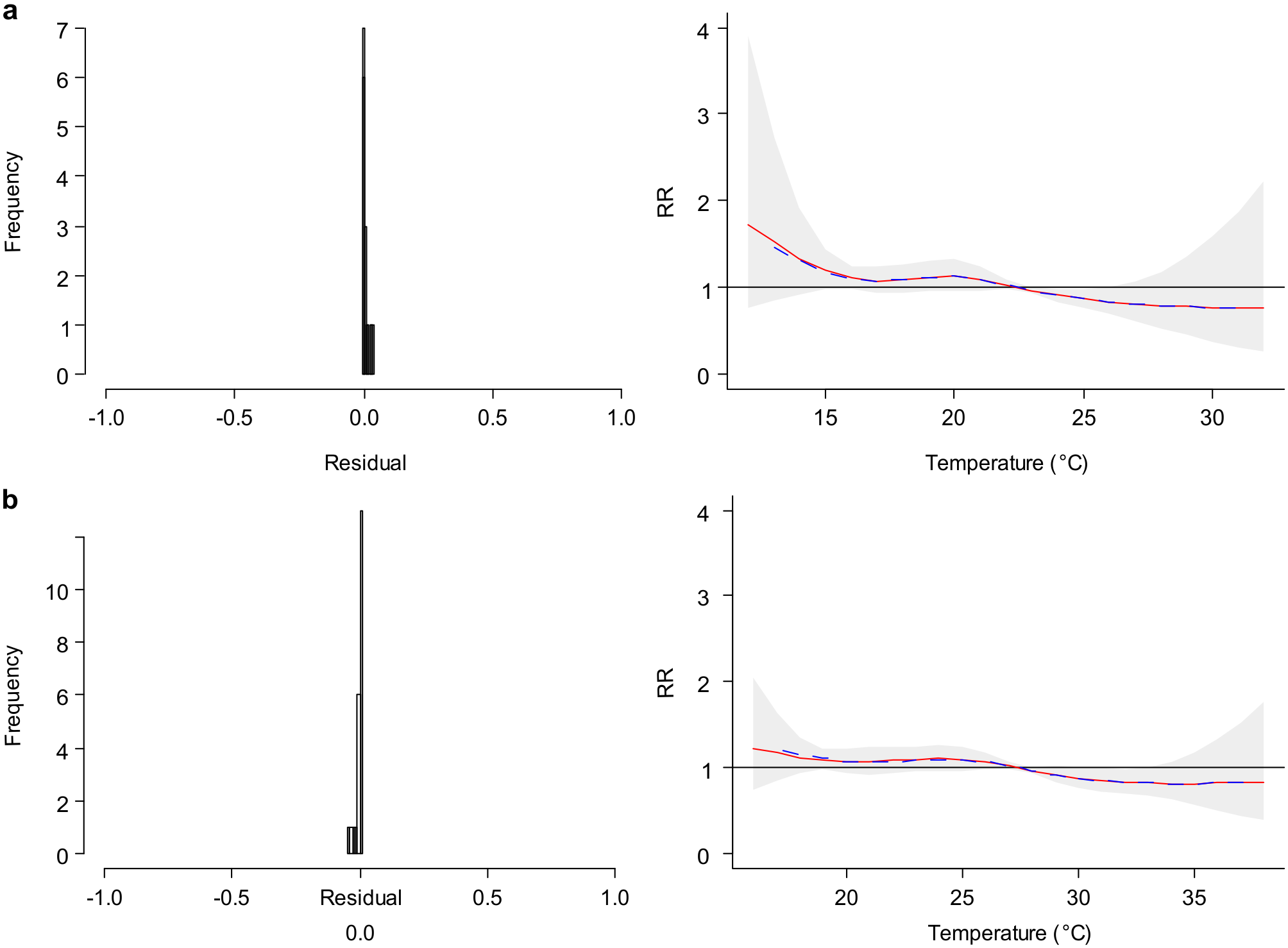

Supplement: Supplementary file 16 — Figure S15. Predictive mortality assessment for simulated data for PMA, 1986–2000 (summer) using 2001–2005 data. DLNM with (a) mean temperature (Tmean), (b) maximum temperature (Tmax). The left panel is the histogram of residuals comparing the risk association prediction for 1986–2000 and 2001–2005, using: (a) mean temperature (Tmean) and (b) maximum temperature (Tmax). The right panel presents the overall cumulative exposure-response association with the red solid line representing the estimates from 1986 to 2000, while the blue dashed line represents the estimates from the 2001–2005. (TIF 271 kb) [file 12940_2019_462_MOESM16_ESM.tif]

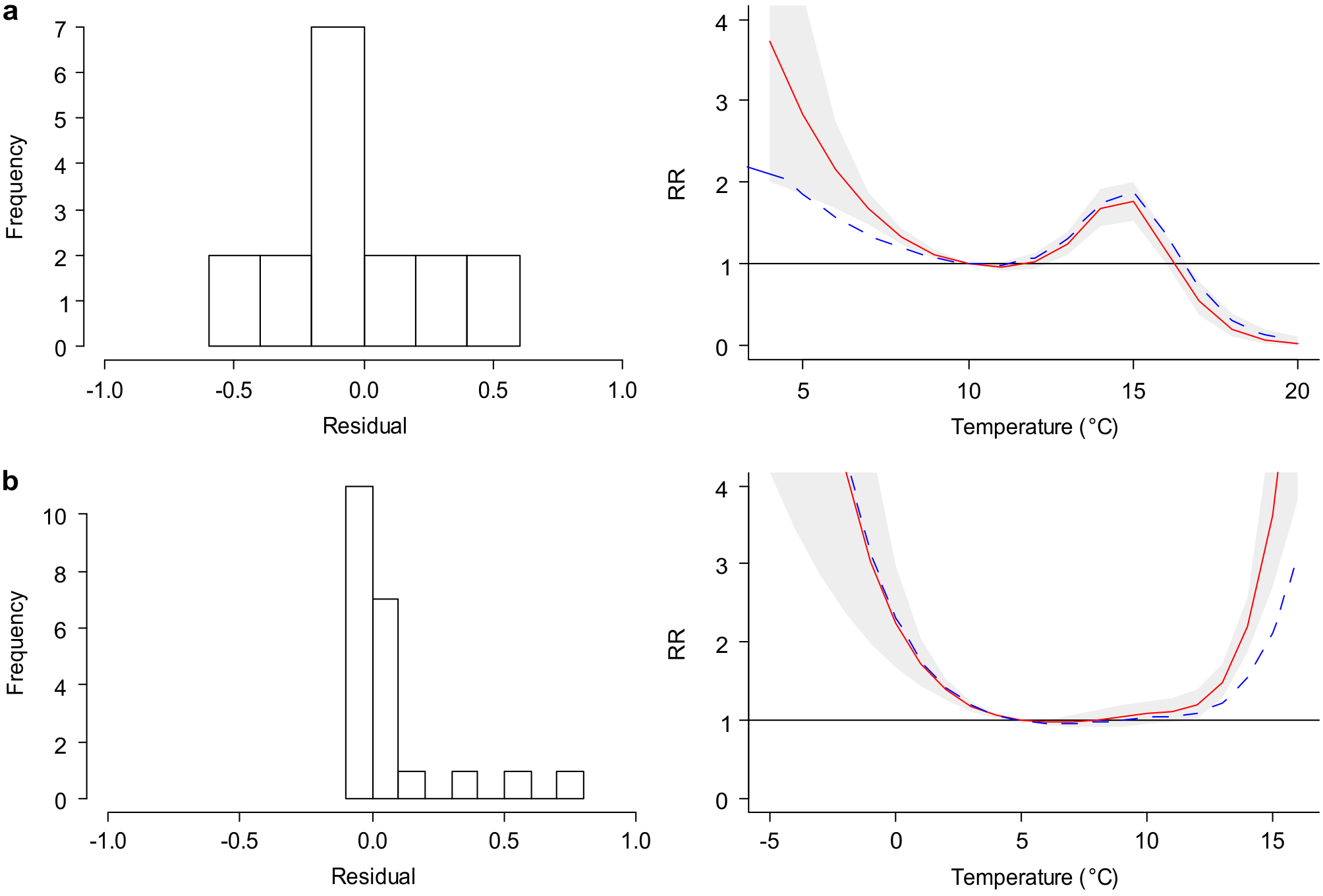

Supplement: Supplementary file 17 — Figure S16. Predictive mortality assessment for simulated data for PMA, 1986–2000 (summer) using 2001–2005 data. DLNM with (a) mean temperature (Tmean), (b) minimum temperature (Tmin). The left panel is the histogram of residuals comparing the risk association prediction for 1986–2000 and 2001–2005, using: (a) mean temperature (Tmean) and (b) minimum temperature (Tmin). The right panel presents the overall cumulative exposure-response association with the red solid line representing the estimates from 1986 to 2000, while the blue dashed line represents the estimates from the 2001–2005. (TIF 297 kb) [file 12940_2019_462_MOESM17_ESM.tif]
