# Supplementary material for: Bootstrap approach to validate the performance of models for predicting mortality risk temperature in Portuguese Metropolitan Areas
Source: Environ Health. 2019 Mar 29;18:25. doi: 10.1186/s12940-019-0462-x (PMC6440075; doi:10.1186/s12940-019-0462-x)
Supplement: Supplementary file 18 — Table S2. Summaries of synthetic indices of relative bias, coverage and relative root mean square error (RMSE) for different parameterization of exposure-lag-response spline function with 8df for DATE to capture trend and seasonality. (DOCX 14 kb) [file 12940_2019_462_MOESM18_ESM.docx]

**Table S2** Summaries of synthetic indices of relative bias, coverage and relative root mean square error (RMSE) for different parameterization of exposure-lag-response spline function with 8 df for DATE to capture trend and seasonality.

| **Model** | **Bias** | **Coverage** | **RMSE** |
| --- | --- | --- | --- |
| Model 1 | 3.9 | 82.6 | 0.36 |
| Model 2 | 3.7 | 80.8 | 0.37 |
| Model 3 | 3.8 | 70.8 | 0.37 |
| Model 4 | 3.9 | 80.6 | 0.36 |
| Model 5 | 3.2 | 81.6 | 0.26 |
| Model 6 | 3.1 | 51.4 | 0.10 |
| Model 7 | 3.8 | 51.4 | 0.10 |
| Model 8 | 3.3 | 51.2 | 0.16 |
| Model 9 | 3.6 | 84.4 | 0.16 |
| Model 10 | 3.1 | 94.4 | 0.07 |
| Model 11 | 1.9 | 94.6 | 0.07 |
| Model 12 | 2.3 | 92.2 | 0.08 |
| Model 13 | 1.7 | 94.4 | 0.16 |
| Model 14 | 1.3 | 82.2 | 0.08 |
| Model 15 | 2.1 | 57.6 | 0.10 |
| Model 16 | 1.9 | 46.4 | 0.16 |
